# Longitudinal Study of the Influence of Periodontal Treatment on the Levels of Insulin Receptor Substrate-2 and Superoxide Dismutase 1 in Individuals with Type 2 Diabetes Mellitus

**DOI:** 10.3390/biomedicines14040742

**Published:** 2026-03-24

**Authors:** François Isnaldo Dias Caldeira, Renata Cristina Lima Silva, Maurício Gandini Giani Martelli, Ingra Gagno Nicchio, Silvana Regina Perez Orrico, Joni Augusto Cirelli, Estela Sasso Cerri, Paulo Sergio Cerri, Fábio Renato Manzolli Leite, Raquel Mantuaneli Scarel-Caminaga

**Affiliations:** 1 Department of Morphology, Genetics, Orthodontics and Pediatric Dentistry, School of Dentistry at Araraquara, UNESP-São Paulo State University, Araraquara 14801-9030, SP, Brazil; francois.isnaldo@unesp.br (F.I.D.C.); renata.cl.silva@unesp.br (R.C.L.S.); mauricio.martelli@unesp.br (M.G.G.M.); ingra.nicchio@unesp.br (I.G.N.); estela.sasso@unesp.br (E.S.C.); paulo.cerri@unesp.br (P.S.C.); 2 Department of Diagnosis and Surgery, School of Dentistry at Araraquara, UNESP-São Paulo State University, Araraquara 14801-9030, SP, Brazil; silvana.orrico@unesp.br (S.R.P.O.); joni.cirelli@unesp.br (J.A.C.); 3 Advanced Research Center in Medicine, Union of the Colleges of the Great Lakes (UNILAGO), São José do Rio Preto 15030-070, SP, Brazil; 4 School of Dentistry, University of Utah, Salt Lake City, UT 84111, USA; fabio@hsc.utah.edu

**Keywords:** periodontitis, type 2 Diabetes Mellitus, IRS2, SOD, inflammation

## Abstract

**Objective**: To longitudinally investigate the effect of non-surgical periodontal therapy (NSPT) on the transcriptional and translational levels of Superoxide Dismutase (SOD) and Insulin Receptor Substrate type 2 (IRS2) in individuals with Type 2 Diabetes Mellitus (T2DM) and Periodontitis (P). **Methods**: This clinical study was registered at the Brazilian Clinical Trials Registry (ReBEC-RBR-5m3yxmb). Saliva, peripheral blood mononuclear cells (PBMCs), and gingival biopsies were collected from 156 individuals, distributed into five groups, each with at least 30 participants: T2DM_poorly_controlled+P, T2DM_well_controlled+P, T2DM_without_P, Periodontitis, and Control. Systemic levels of messenger RNA (mRNA) of Superoxide Dismutase 1 (SOD1) and IRS2 were measured using real-time polymerase chain reaction at baseline, 90, and 180 days after NSPT. SOD enzymatic activity in Saliva and IRS-2 immunohistochemistry in gingival biopsies were also assessed. **Results**: Higher *SOD1* mRNA levels were observed in Control individuals at baseline. In contrast, higher *IRS2* mRNA levels were detected in individuals with Periodontitis at baseline, followed by a significant reduction over time. A significant positive longitudinal correlation was identified between IRS2 and SOD1 gene expression in the groups without T2DM, indicating potential functional interaction between the molecules. Salivary SOD enzymatic activity was lower in individuals from the T2DM_poorly_Controlled+P and T2DM_well_Controlled+P groups. SOD concentration (U/g) normalized to the total protein content was higher in the saliva of individuals with Periodontitis. T2DM+P and Periodontitis groups showed extensive inflammatory infiltrate in the gingival biopsies, with predominant IRS-2 immunopositive cells in the T2DM+P groups, independently of the metabolic control. **Conclusions**: This study shows that non-surgical periodontal therapy (NSPT) is followed by longitudinal changes in IRS2 and SOD1 expression at the mRNA and protein levels in individuals with T2DM+P (poorly/well controlled) and periodontitis, reinforcing the clinical relevance of periodontal treatment in the systemic context of T2DM.

## 1. Introduction

Periodontitis is an inflammatory disease characterized by the progressive destruction of the supporting tissues of the teeth, with clinical manifestations varying due to the host’s immune and bacterial responses [1,2]. Understanding the multidimensional aspects of this disease poses a significant challenge for healthcare professionals, given that periodontitis is considered one of the leading causes of tooth loss in the adult population [3].

Type 2 Diabetes Mellitus (T2DM) is a chronic metabolic disease and a major global health problem for healthcare systems [4,5]. Recognized as a metabolic disorder, it is characterized by persistent hyperglycemia. T2DM has been recognized as a complex and multifactorial disease that significantly affects individuals’ lifestyles [6]. Reduced effectiveness of insulin action in tissues is defined as insulin resistance [7].

Glycated hemoglobin (HbA1c) is a biochemical method used to measure blood glucose levels and is commonly employed in clinical trials as a marker of metabolic control [8]. It is considered the most reliable and reproducible method for predicting the level of glycemic control and its associated complications. Individuals with HbA1c levels ≤7% (<53 mmol/mol) tend to present more favorable conditions and a more predictable response to treatment [8,9]. Conversely, individuals with HbA1c levels >7% (>53 mmol/mol) are considered outside the metabolic target and usually exhibit less favorable outcomes, particularly in nonsurgical periodontal treatment [8].

Periodontitis has gained prominence as the sixth complication associated with T2DM. Numerous epidemiological [10], clinical [11,12], and genetic studies [13,14] have attempted to understand the bidirectional relationship between these two diseases [15,16,17]. Furthermore, investigations have analyzed the effects of these conditions on the transcriptional and translational levels of micro and macromolecules using various analytical methods [18,19,20].

The insulin receptor substrate 2 (IRS2) plays a crucial role as a regulator of glucose homeostasis, participating in various metabolic and mitogenic functions [21]. Together with IRS1, IRS2 is responsible for signal transduction, activating the phosphatidylinositol 3-kinase and protein kinase B (PI3K-PKB) pathway [22]. This activation leads to the translocation of vesicles containing glucose transporter type 4 (GLUT4) to the plasma membrane, facilitating glucose influx into the intracellular space. Additionally, these substrates trigger a positive signaling cascade, activating NF-E2-related factor 2 and Kelch-like ECH-associated protein (Nrf2-Keap1), which are responsible for the production of antioxidant enzymes, including superoxide dismutase (SOD) [23].

Superoxide dismutases are a group of enzymes involved in intracellular detoxification processes that play essential roles in homeostasis [24]. SOD catalyzes the reduction of superoxide radicals such as O_2_•− (superoxide radical anion) and hydrogen peroxide (H_2_O_2_). Furthermore, other enzymes such as glutathione peroxidase (GPx) and catalase accelerate the reduction of H_2_O_2_ to water [25]. Under normal biological conditions, there is a balance between the antioxidant defense system and reactive oxygen species (ROS) [18].

In patients affected by T2DM and Periodontitis, homeostatic signaling pathways have become altered or compromised. In the context of hyperglycemia, insulin receptor (IR) phosphorylation at serine or threonine residues leads to the inactivation of tyrosine kinase capacity, resulting in reduced PI3K-PKB action, GLUT4 translocation, and decreased antioxidant system activity [22,26]. However, other pathways are activated, such as c-Jun N-terminal kinase-Activator Protein-1 (JNK-AP-1) and I kappa B kinase-Nuclear factor kappa-B (IKK-NF-kB), contributing to increased production of ROS [18] and pro-inflammatory cytokines, highlighting tumor necrosis factor-alpha (TNF-α) [21].

There are various gaps in the literature regarding the physiological role of antioxidants and glycemic metabolism in individuals simultaneously affected by T2DM and Periodontitis, as well as the extent to which IRS2 and SOD might be modulated by non-surgical periodontal therapy (NSPT). In particular, studies assessing IRS2 and SOD1 simultaneously in a longitudinal design after NSPT, and integrating systemic and oral biological matrices, remain limited. Therefore, the objective of this study was to investigate the effect of NSPT on the transcriptional and translational levels of IRS2 and SOD1 in individuals simultaneously affected by these pathologies. Additionally, we aimed to perform correlation analyses between the investigations of molecular outcomes (IRS2 and SOD1 indicators) and relevant clinical and biochemical variables. Besides a deeper understanding of the biological interplay of these molecules, this study could contribute evidence on whether NSPT is followed by measurable changes in IRS2 and SOD1 across different sample types, supporting their investigation in the context of T2DM–periodontitis comorbidity [27]. To achieve this, we utilized peripheral blood mononuclear cells (PBMCs) to assess the gene expression of IRS2 and SOD1, considering that the circulating expression of these genes reproduces systemic alterations occurring in both diseases. Additionally, we conducted immunolocalization analyses of IRS-2 in human gingival tissue biopsies and assessed the enzymatic activity of SOD in saliva to provide complementary evidence at the gene, protein, and functional levels and to determine whether NSPT is accompanied by changes in IRS-2 and SOD1 across systemic and local compartments. Null hypothesis: NSPT does not induce significant changes in IRS2 and SOD1 transcriptional and translational levels, and no significant correlations exist between IRS2/SOD1 indicators and the investigated clinical or biochemical variables.

## 2. Materials and Methods

To enhance the quality of the scientific writing of the study, the TREND (Transparent Reporting of Evaluations with Nonrandomized Designs) checklist was employed [28]. The individuals included in this study, after being informed about the objectives and methods, provided their written consent to participate in this study. The study was conducted from January 2021 to December 2023 in accordance with the Helsinki Declaration of 1975 and Brazilian regulations 466 (2012) for research involving human subjects. The study protocol received approval from the Research Ethics Committee with Human Beings of the School of Dentistry at Araraquara, São Paulo State University, Brazil (Protocol number CAAE-44639821.8.0000.5416). This clinical study was registered at the Brazilian Clinical Trials Registry (ReBEC) with the identification number RBR-5m3yxmb. The date of first recruitment recorded in the registry is 1 July 2021.

### 2.1. Sample Size Calculation and Researcher Calibration

This study was designed to assess the impact of the NSPT on reductions in periodontal pockets ≥ 6 mm, as well as its effect on the gene expression and protein levels of IRS2 and SOD. The sample size calculation was assessed by the G*Power Calculator, version 3.1.9 [29], considering the following parameters: ANOVA: repeated measures, between factors; α = 0.05 and 80% power. It was found that each group should contain at least 27 subjects to detect associations between IRS2 and SOD expression with T2DM and Periodontitis. More information on participant calibration can be found in Supplementary Materials S1.

### 2.2. Study Population

Individuals were recruited from the Diagnosis and Surgery Department of the School of Dentistry at Araraquara, São Paulo State University (FOAr-UNESP). Participants were of both genders, any ethnicity, aged between 30 and 65 years, and had a minimum of 15 teeth. Medical and dental records were obtained to determine the oral and systemic health conditions of the participants.

The diagnosis of T2DM was made by an endocrinologist who monitored the glycemic levels of each participant by evaluation of glycated hemoglobin (HbA1c) values, according to the American Academy of Diabetes (American Diabetes Association Professional Practice, 2022) [30]. Further information can be found in Supplementary Materials S1.

**Figure 1:**
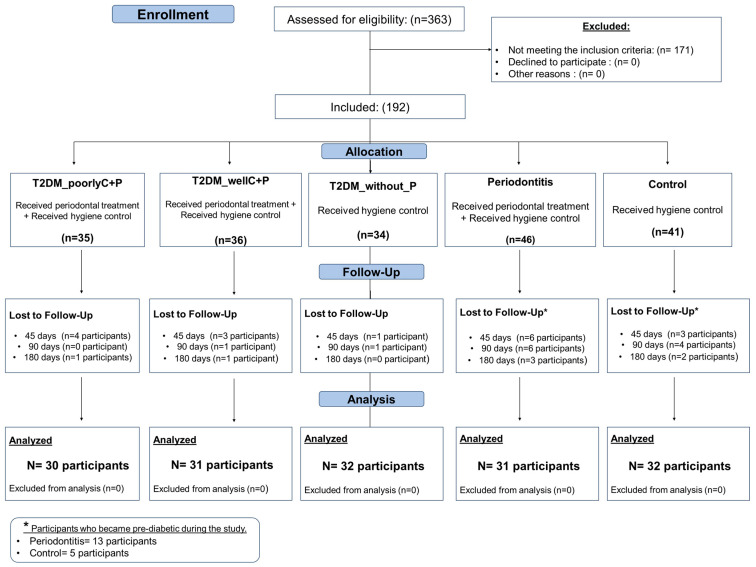
Flowchart of participant allocation for the study.

Individuals with a history of hepatitis and HIV infection, anemia, antibiotic premedication in the last 6 months, chronic use of anti-inflammatory drugs, pregnant or lactating women, and prediabetic individuals were excluded from the study. Figure 1 shows the flowchart of the subjects’ recruitment, allocation into the groups, and follow-up. Thus, 156 individuals were included in the study and distributed across five experimental groups: Control (n = 32), Periodontitis (P) (n = 31), T2DM_without_P (n = 32), T2DM_wellC+P (n = 31), and T2DM_poorlyC+P (n = 30).

### 2.3. Physical and Biochemical Evaluations

Physical examinations were conducted to assess abdominal circumference, waist-to-hip ratio, weight (kg), and height (m) for the calculation of Body Mass Index (BMI) in kg/m^2^. Biochemical parameters were assessed at baseline and follow-up by referring each subject (after a 10–12-h overnight fasting) to a clinical analysis laboratory that collected a blood sample for evaluating insulin levels by the chemiluminescence method (U/L), fasting plasma glucose (mg/dL) by the modified Bondar & Mead method, and HbA1c by enzymatic immunoturbidimetry. The homeostasis model assessment (HOMA) was evaluated to calculate insulin resistance (IR). The Total Cholesterol (TC), High-density lipoprotein Cholesterol (HDL-Cholesterol), and Triglycerides were measured by enzymatic methods. The fractions of low-density lipoprotein cholesterol (LDL-Cholesterol) and very-low-density lipoprotein (vLDL) were calculated according to the Friedewald equation [31]. Non-HDL-Cholesterol was calculated as Non-HDL-Cholesterol = TC-HDL, with the abnormal cutoff value ≥ 130 mg/dL, which is considered to be a good predictor of cardiovascular disease (CVD) risk [32].

### 2.4. Periodontal Evaluations

The following periodontal parameters were assessed at the baseline and follow-up study: Visible Plaque Index (VPI), Marginal Bleeding Index (MBI), Bleeding on Probing (BoP), Probing Depth (PD), Gingival Margin Position, and Clinical Attachment Loss (CAL). These parameters were examined at six sites (mesio-buccal, buccal, disto-buccal, mesio-lingual, lingual, and disto-lingual) of each tooth, using a sterilized millimeter-marked Williams-type periodontal probe (Trinity^®^—Campo Mourão, Paraná, Brazil) and mouth mirror. To be considered affected by Periodontitis, individuals were required to have generalized Periodontitis in stages II or III and grades A, B, or C, with a minimum of 6 non-adjacent proximal sites on different teeth, with PD ≥ 5 mm, CAL ≥ 4 mm, and the presence of BoP. Periodontally healthy controls were required to have PD ≤ 3 mm and BoP in less than 10% of sites [33,34].

Periodontal treatment was performed according to the needs of each individual. For periodontally healthy patients, only prophylaxis with a rubber cup and prophylactic paste was carried out. For individuals with Periodontitis, the NSPT was conducted by a single trained professional [35,36]. More information on NSPT can be found in Supplementary Materials S1.

### 2.5. Reverse Transcription—Quantitative Real-Time Polymerase Chain Reaction (RT-qPCR)

During blood collection for biochemical assays, an additional tube containing EDTA/K3 as an anticoagulant was collected for isolating peripheral blood mononuclear cells (PBMCs) to extract RNA for gene expression evaluation. PBMCs were isolated using a density gradient with Histopaques (1077 and 1119, Sigma-Aldrich, St. Louis, MO, USA). After centrifugation, PBMCs from each sample were obtained, homogenized in TriZol (ThermoFisher Scientific, Waltham, MA, USA), and stored in −80 °C ultrafreezer. The total RNA from PBMC was extracted following the TriZol (ThermoFisher Scientific) protocol. The purity and quantity of RNA were assessed using a microvolume UV spectrophotometer (Nanovue Plus, GE Health Sciences, Little Chalfont, Buckinghamshire, UK). cDNA synthesis was performed using 460 ng of total RNA utilizing the High-Capacity RT kit (ThermoFisher Scientific). Real-time qPCR was conducted using pre-designed primer and probe sets (gene expression assays, ThermoFisher Scientific) for the detection of target genes (*IRS2*, *SOD1*) and constitutive genes (*GAPDH*, *ACTG*—Table S1, Supplementary Materials) through the TaqMan system (TaqMan Universal PCR Master Mix, ThermoFisher Scientific) utilizing the StepOne Plus equipment (ThermoFisher Scientific), following cycling conditions optimized by the manufacturer. The Cycle threshold (Ct) value of each target gene was normalized considering the mean of constitutive genes, calculated by ThermoFisher Scientific Cloud, and analyzed using the 2^−ΔCt^ method.

### 2.6. Salivary Protein Quantification and Evaluation of the Enzymatic Activity of SOD

Unstimulated saliva sample was obtained from all participants by freely expectoration of saliva for 5 min into a 15 mL Falcon tube on ice. All samples were centrifuged at 4500× *g* for 20 min at 4 °C to discard the pellet and separate the supernatant into aliquots, which were subsequently stored at −80 °C.

The quantification of total protein in each saliva sample was carried out by the Bicinchoninic Acid method using the BCA protein assay (BCA1, Sigma-Aldrich). The SOD activity was assessed using the Ransod kit (SD125—Randox, Co., Antrim, UK). This method employs xanthine-xanthine oxidase (XOD) to generate superoxide radicals that react with 2-(4-iodophenyl)-3-(4-nitrophenol)-5-phenyltetrazolium chloride (I.N.T.), forming the red formazan compound. SOD activity was measured by the degree of inhibition of this reaction. Five dilutions of the standard protein (6.55 U/mL, 3.27 U/mL, 1.63 U/mL, 0.81 U/mL, 0.40 U/mL) were prepared to construct the standard curve. Initial and final absorbances were determined at a wavelength of 505 nm. The concentration results of SOD were expressed in U/mL of saliva. SOD activity (U/g) was normalized by the total protein quantified by the BCA method.

### 2.7. Immunohistochemistry of Gingival Biopsies

A gingival tissue fragment was obtained from individuals who required additional surgical procedures following the NSPT. All clinical characteristics of the donor sites were noted (BoP, PD, and CAL). The removed tissue generally had a thickness of about 1 mm, which could be greater based on surgical indications. Immediately after removal, the tissue was briefly washed in physiological saline and placed in a 4% formaldehyde solution containing 0.1 M sodium phosphate with a pH of 7.2 for 48 h. After fixation, the fragments were dehydrated and embedded in paraffin. Sections of 5-μm thick were obtained from each specimen, adhered to silanized slides, and stained with hematoxylin and eosin (H&E) for morphological and quantitative analyses.

The immunohistochemical reaction for gingival tissue involved deparaffinization and antigen retrieval by immersing the slides in 0.001 M sodium citrate buffer at pH 6.0 and heating in a microwave oven (1 cycle at maximum microwave power until boiling; 2–5 min at power 180 W; 2–5 min at power 90 W; and 2–5 min at power 90 W). After cooling, the endogenous peroxidase activity was inhibited using a 5% hydrogen peroxide solution, and non-specific binding was blocked with a 5% bovine serum albumin (BSA, Sigma, St. Louis, MO, USA) solution in PBS. The samples were incubated overnight with the rabbit primary antibody against IRS-2 (AB Clonal—A7945, Woburn, MA, USA), following the manufacturer’s instructions, at a concentration of 1:150. As a negative control, the primary antibody was omitted, and the slides were incubated with 1% BSA to assess background staining. The samples were extensively washed, and subsequently the sections were incubated for 1 h in goat-rabbit HRP conjugate (Detection Kit Micro-polymer, Abcam ab236466, Cambridge, UK) at room temperature. After washing, the reaction was revealed with 3.3’-diaminobenzidine (Abcam) and counterstained with Carazzi’s hematoxylin.

In each section, approximately three standardized fields of the lamina propria from the gingival biopsy were captured at ×1450 magnification. The total number of IRS2-positive cells in each field was quantified by a masked, calibrated examiner. The numerical density of immunolabeled cells was then calculated by dividing the total number of immunostained cells by the corresponding standardized area. For comparative statistical analysis, an average IRS2-positive cell density value was subsequently obtained for each specimen within each group.

### 2.8. Data Analysis

Data were analyzed using GraphPad Prism 8.0.1 (GraphPad Software Inc., San Diego, CA, USA) with α = 0.05. Normality was assessed using the Shapiro–Wilk test. Categorical demographic variables (sex, skin color, and smoking) were compared using the Chi-square test. Continuous variables were compared among groups using one-way ANOVA with Tukey’s post hoc test (parametric) or the Kruskal–Wallis test with Dunn’s post hoc test (nonparametric). Longitudinal comparisons (baseline, 90, and 180 days) were performed using repeated-measures one-way ANOVA with Tukey’s test (parametric) or the Friedman test with Dunn’s test (nonparametric). Correlations were assessed using Spearman’s test (clinical/biochemical vs. gene expression) and Pearson’s test (gene–gene).

## 3. Results

### 3.1. Participant Allocation

A total of 363 participants were recruited for screening and analysis of the study’s inclusion criteria. One hundred and seventy-one participants did not meet the criteria established by the study. Thus, 192 individuals effectively participated in the study (Figure 1). During the non-surgical periodontal treatment, there was a loss of 19 participants, along with the need to replace 17 participants belonging to the Periodontitis and Control groups who became pre-diabetics. Finally, the longitudinal study included 156 individuals distributed into five groups: T2DM_poorlyC+P = 30, T2DM_wellC+P = 31, T2DM_without_P = 32 (T2MD_poorlyC_withoutP = 18 and T2DM_wellC_withoutP = 14), Periodontitis = 31, and Control = 32.

### 3.2. Demographic, Physical, Biochemical, and Periodontal Parameters

The demographic and physical data are presented in Table 1. Each group was composed mainly of never-smokers, highlighting that in the Control group, there were no former-smokers, leading to the significant difference in its distribution among groups (*p* = 0.003).

The participants’ biochemical results can be seen at baseline and follow-up in Table 2. The T2DM_poorlyC+P group had the highest levels of HbA1c and insulin resistance (HOMA-IR index) compared to the others, which did not show a reduction during longitudinal follow-up (intra-group analysis). In the longitudinal analysis, no significant differences were observed in plasma glucose, insulin, and HOMA-IR data in any group. In the LDL cholesterol index, a statistically significant reduction (*p* = 0.0239) was observed in the T2DM_poorlyC+P group between 90 and 180 days. Triglyceride levels showed statistically significant differences among groups in all periods.

When analyzing the data from the groups with periodontitis, higher levels of BoP were observed, which were significantly different from the groups without periodontal disease (*p* = 0.0001). All groups showed a significant decrease in BoP during longitudinal follow-up. Regarding CAL, a significant improvement was observed in both T2DM_poorlyC+P and T2DM_wellC+P groups longitudinally (baseline-180 days and baseline-90 days; *p* < 0.0001).

### 3.3. Gene Expression

**Figure 2:**
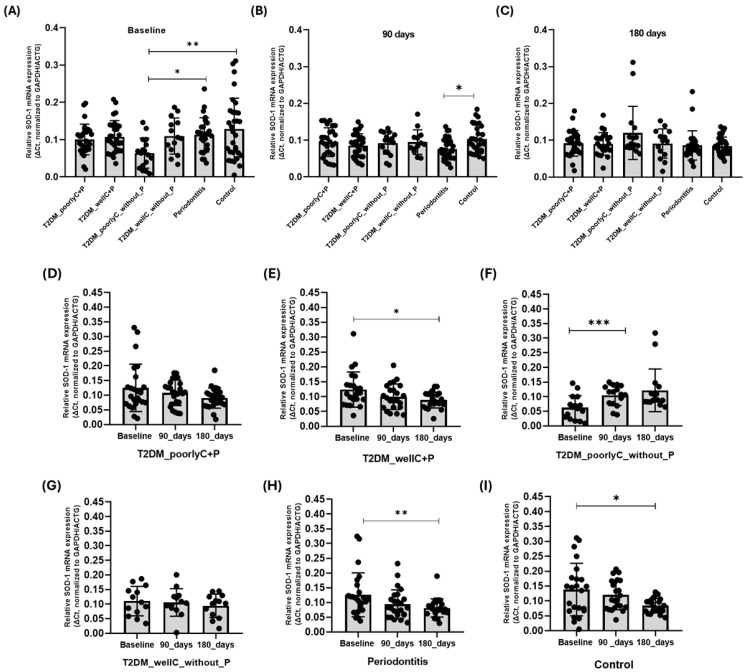
*SOD1* mRNA expression levels in the PBMC of participants: (**A**) baseline, (**B**) 90 days, (**C**) 180 days, *SOD1* mRNA expression levels in PBMCs of participants: (**D**) T2DM_poorlyC+P, (**E**) T2DM_wellC+P, (**F**) T2DM_poorlyC_without_P, (**G**) T2DM_wellC_without_P (**H**) Periodontitis (**I**) Control. T2DM_poorlyC+P= Type 2 Diabetes Mellitus_poorly controlled+Periodontitis; T2DM_wellC+P = Type 2 Diabetes Mellitus_well controlled+Periodontitis; T2DM_poorlyC_without_P = Type 2 Type 2 Diabetes Mellitus_poorly controlled_without Periodontitis; T2DM_wellC_without_P = Type 2 Type 2 Diabetes Mellitus_well controlled_without Periodontitis. Groups were compared using the One-way ANOVA test, followed by Tukey’s post-test; * = *p* < 0.05; ** = *p* < 0.001; *** = *p* < 0.0001. One-way ANOVA was used to compare baseline, 90, and 180 days, followed by Tukey’s post-test.

For this analysis, we subdivided T2DM_without_P according to the HbA1c levels of the individuals, forming the T2DM_poorly_controlled_without_P and T2DM_well_controlled_without_P. Figure 2 shows the PBMC mRNA expression levels of *SOD1* at baseline and follow-up. At baseline, we observed that there was a significantly higher expression of *SOD1* in individuals affected only by Periodontitis compared to those with the T2DM_poorlyC_withoutP group (*p* = 0.044). Additionally, we noted the highest expression of *SOD1* in the Control group, which was significantly different from the T2DM_poorlyC_withoutP group (*p* = 0.001) (Figure 2A). After 90 days of the NSPT, we observed higher expression of *SOD1* in the Control group compared to the group with only Periodontitis (*p* = 0.0311) (Figure 2B).

In the longitudinal evaluation of each group, we found that participants with periodontal disease showed a gradual decrease in gene expression, with *SOD1* mRNA levels decreasing from baseline to the end of NSTP (Figure 2H). The T2DM_wellC+P (Figure 2E), Periodontitis (Figure 2H), and Control (Figure 2I) groups showed statistically significant reduction of *SOD1* mRNA expression from baseline to 180 days (*p* = 0.016, *p* = 0.003, and *p* = 0.0112, respectively).

**Figure 3:**
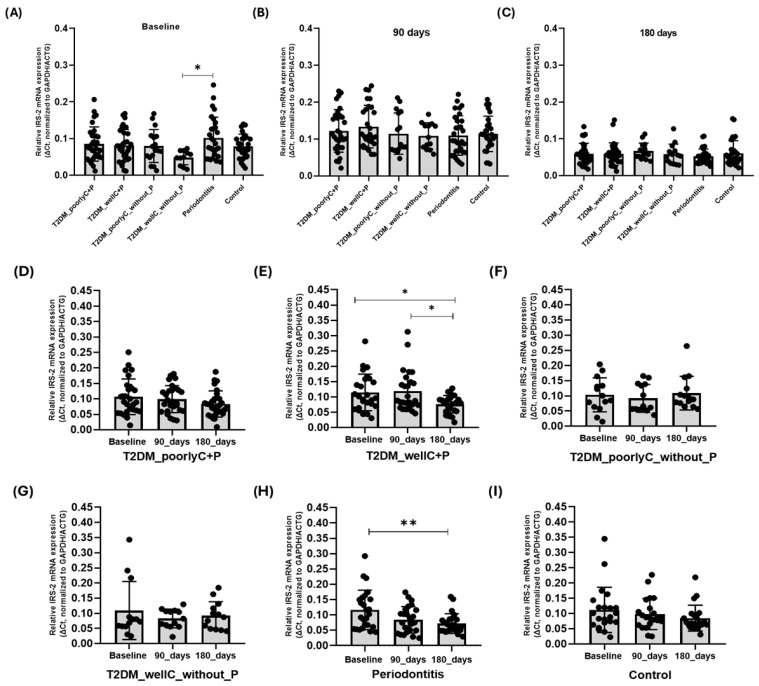
*IRS2* mRNA expression levels in the PBMC of participants: (**A**) baseline, (**B**) 90 days, (**C**) 180 days, *IRS2* mRNA expression levels in PBMCs of participants: (**D**) T2DM_poorlyC+P, (**E**) T2DM_wellC+P, (**F**) T2DM_poorlyC_without_P, (**G**) T2DM_wellC_without_P (**H**) Periodontitis (**I**) Control. T2DM_poorlyC+P = Type 2 Diabetes Mellitus_poorly controlled+Periodontitis; T2DM_wellC+P = Type 2 Diabetes Mellitus_well controlled+Periodontitis; T2DM_poorlyC_without_P = Type 2 Type 2 Diabetes Mellitus_poorly controlled_without Periodontitis; T2DM_wellC_without_P = Type 2 Type 2 Diabetes Mellitus, well controlled_without Periodontitis. The groups at baseline, 90, and 180 days were compared using the one-way ANOVA test, followed by Tukey’s post-test. * = *p* < 0.05; ** = *p* < 0.001. Friedman’s non-parametric test was used, followed by Dunn’s post-test for all longitudinal groups.

Regarding the *IRS2* gene expression, at baseline, participants with Periodontitis expressed significantly higher levels of *IRS2* than those from the T2DM_wellC_withoutP group (*p* = 0.013) (Figure 3A). Regarding the participants in the Periodontitis group, it was interesting to note a gradual decrease in *IRS2* gene expression, with a statistically significant difference between baseline and 180 days after NSPT (*p* = 0.011) (Figure 3H).

**Figure 4:**
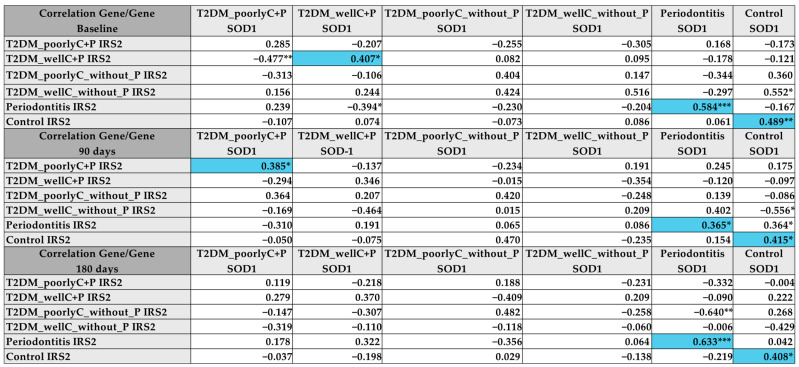
Correlation between *SOD1* and *IRS2* mRNA levels in PBMC of the participants. The values in the table are described in ρ. Spearman correlation. Blue-colored bills indicate a significant and directly proportional correlation. * *p* < 0.05; ** *p* < 0.01; *** *p* < 0.001.

Furthermore, in gene-gene correlation analyses, we identified a significant positive (moderate to strong rating) association between the SOD1 and IRS2 gene expressions at baseline for T2DM_wellC+P (ρ = 0.407) and at 90 days in the T2DM_poorlyC+P group (ρ = 0.385). Interestingly, we observed a significant longitudinal correlation between IRS2 and SOD1 genes in the groups without T2DM (Figure 4). Significant longitudinal direct correlation was identified between IRS2 and SOD1 gene expression in the groups without diabetes (Control and Periodontitis), indicating potential functional interaction between the molecules.

Correlation analyses were also performed to investigate potential relationships among the systemic gene expression of IRS2 and SOD1, SOD enzyme activity, physical, biochemical, and periodontal parameters in each group and the studied period (Supplementary Figures S1–15). Significant correlation findings (inversely proportional associations) were observed, such as for the T2DM_poorlyC+P group at baseline, between HOMA-IR and *IRS2* gene expression (ρ = −0.291; *p* = 0.04), as well as HOMA-IR and *SOD1* gene expression (ρ = −0.469; *p* = 0.005). In the Periodontitis group at baseline, there was a significant inverse correlation between BoP and the gene expression of *SOD1* (ρ = −0.410; *p* = 0.022) and *IRS2* (ρ = −0.535; *p* = 0.002); and also, between gene expression of *SOD1* and CAL ≥ 5 mm (ρ = −0.387; *p* = 0.031). Some periodontal parameters were correlated with physical or biochemical parameters, for example, in Periodontitis at baseline, we found CAL ≥ 5 mm correlated with BMI (ρ = 0.369; *p* = 0.047). In the follow-up, we highlight the inverse proportional correlations of CAL ≥ 5 mm with total cholesterol, HDL, LDL, and Non-HDL-Cholesterol (Supplementary Figures S6–15).

### 3.4. Protein Expression

For this analysis, we subdivided T2DM_without_P into two HbA1c metabolic controls (T2DM_poorly_controlled_without_P and T2DM_well_controlled_without_P). The baseline, when analyzing SOD concentration in saliva of subjects, showed that the T2DM_wellC+P group had lower levels of SOD concentration (U/mL) compared to the T2DM_poorlyC_withouth_P group (*p* = 0.0459) and the Control group (*p* = 0.0273, Table 3). When analyzing the longitudinal data, we observed a gradual increase in SOD concentration (U/mL) in all investigated groups.

Alternatively, when normalizing SOD concentration (U/mL) by the total amount of protein present in saliva (quantified using the BCA method), the results indicated the SOD activity (U/g), which was higher at baseline in the T2DM_poorlyC+P group, as well as in the Periodontitis group compared to the Control group (respectively *p* = 0.0013, and *p* = 0.009) (Table 3). In the longitudinal analysis, many groups exhibited a significant increase in salivary SOD (U/g) activity.

**Figure 5:**
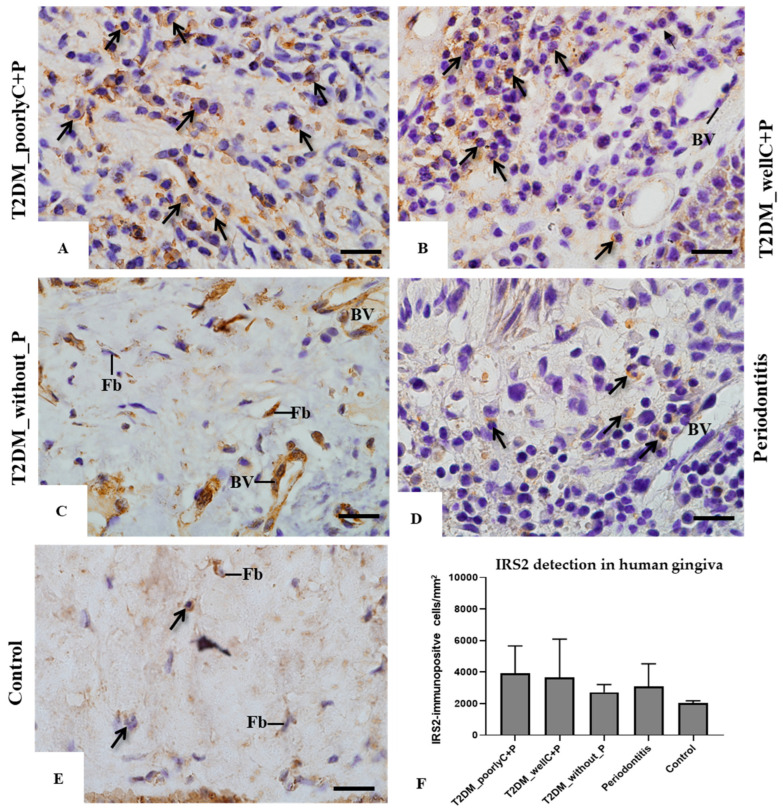
Light micrographs of portions of human gingival biopsies submitted to immunohistochemistry for IRS detection and counterstained with hematoxylin. Light micrographs of human gingiva showing portions of the lamina propria from T2DM_poorlyC_P (**A**), T2DM_wellC_P (**B**), T2DM_without_P (**C**), Periodontitis (**D**), and Control (**E**) groups. The sections were subjected to immunohistochemistry for the detection of IRS-2 (brown-yellow color) and counterstained with hematoxylin. Arrows: inflammatory cells; Fb: fibroblasts; BV: blood vessel. Scale bars: 20 µm. Figure (**F**): number of IRS-2-immunolabelled cells in the lamina propria of human gingiva biopsies.

Furthermore, we assessed the inflammatory content and immunolocalization of IRS-2 in gingival tissue biopsies. The histological sections illustrated in Figure 5 revealed distinct characteristics for each group: in the T2DM_poorlyC+P group, we observed a large inflammatory infiltrate composed mainly of neutrophils, macrophages, and plasma cells (neutrophils and eosinophils) and staining of IRS-2 throughout the cell cytoplasm (in light brown). For the T2DM_wellC+P group, besides the significant inflammatory infiltrate, we noted a slightly more intense IRS-2 immunolabelled cell cytoplasm than the T2DM_poorlyC+P group. In the T2DM_without_P group, besides low inflammatory infiltrate, the IRS-2 immunostaining was more circumscriptive to the endothelium. In the Periodontitis group, there was an intense inflammatory infiltrate, as was observed in the T2DM_poorly/wellC+P, but showing a lower number of IRS-2-immunolabeled cells. In the Control group, the absence of inflammatory cells and higher content of amorphous material in the lamina propria showed rare fibroblasts IRS-2-immunolabelled.

## 4. Discussion

Numerous epidemiological [9,10], clinical [11,12], and genetic studies [13,14] have attempted to understand the bidirectional relationship between T2DM and Periodontal diseases [15,16,17,37]. To our knowledge, this is the first study to evaluate the relationship between IRS-2 (an important molecule for insulin signal transduction) and SOD (an enzyme crucial for the balance of the oxidant-antioxidant system) in individuals simultaneously affected by T2DM and periodontitis before and after NSPT.

The results presented here demonstrated that the null hypothesis was rejected. Primary findings of our study indicated that NSPT had a positive impact on the periodontal status of the individuals, improving BoP and PD indices of groups with periodontitis. These results interestingly align with previous research that also demonstrated the short-term impact and benefits of NSPT on periodontal clinical parameters in T2DM patients [38,39]. The effects of this therapy on biochemical parameters, mainly HbA1c, were evaluated. Surprisingly, our results showed no significant HbA1c level reduction in the follow-up of NSPT for any group. In this context, a clinical study conducted in Turkey involving patients with Periodontitis and T2DM also did not identify a significant reduction in HbA1c levels as an effect of NSPT [40]. These findings were consistent with a Japanese and German study that investigated the short-term effect of this therapy on HbA1c levels and inflammatory markers in T2DM patients [38].

Phosphorylation of tyrosine residues of IRS1/IRS2 intensifies insulin signal transduction [41], while insulin-resistant patients have elevated levels of interleukin 6 (IL-6) and TNF-α, resulting in the activation of JNK-AP-1 and IKK-NF-kB pathways that phosphorylate IRS1/IRS2 on serine residues, inhibiting insulin signaling [21,42]. To the best of our knowledge, this is the first study to demonstrate the increased systemic expression of the *IRS2* gene in subjects with periodontitis compared to well-controlled T2DM individuals without periodontitis. A possible explanation for the significant increase in IRS2 mRNA expression in patients with periodontitis may be attributed to the dysregulation of the inflammatory response [43], as well as the elevation in levels of pro-inflammatory markers present both locally and systemically in individuals with periodontitis [21].

Moreover, for Periodontitis patients’ mRNA, *IRS2* levels significantly decreased after NSPT, reaching normal levels compared to the Control group. This might suggest that the NSPT could contribute to the regulation of the transduction mechanism of insulin signaling [41]. The qualitative and quantitative findings of the gingival biopsies IRS-2 immunolabelled demonstrated a reduction of IRS-2 staining according to the low evidence of inflammation, since the gingival lamina propria of Controls showed rare fibroblasts IRS-2 immunolabelled, contrasting with the intense presence of polymorphonuclear cells expressing IRS-2 in the inflammatory infiltrate in T2DM+P patients.

Analyzing the correlation results of our study, we observed significant correlations between physical, biochemical, and periodontal parameters (Supplementary Materials). This agrees with a study that indicated a positive correlation between increased body fat, insulin resistance, and the prevalence of periodontitis [44]. The mechanisms behind these conditions are believed to involve altered insulin sensitivity, increased oxidative stress, and the formation of advanced glycation end products [45].

Current literature has highlighted the relationship between the imbalance of the antioxidant-oxidant system and the progression of periodontitis. Antioxidant enzymes play a crucial role in catalyzing superoxide radicals and protecting cells from ROS [46]. In the present study, the comparison between the baseline and follow-up of the study showed a significant reduction in systemic mRNA expression of *SOD1* in individuals with Periodontitis (after NSPT). Interestingly, a study conducted by Ferreira et al. 2023 identified higher levels of *SOD1* mRNA in control patients [27]. However, a study on gingival tissue samples showed a higher trend of *SOD1* expression in patients with T2DM and Periodontitis [37].

Analyzing the correlation findings of our study, we observed an inverse proportional association between *SOD1* mRNA levels and Body Mass Index (BMI) in T2DM_poorlyC+P. Furthermore, there is evidence that in obese patients with periodontitis, neutrophils become more hyperactive, leading to increased cytoplasmic production of superoxides and inflammatory mediators [45,47,48]. In this context, our correlation results between physical parameters and *SOD1* gene expression suggest a potential interaction among the glycemic parameters observed for T2DM, periodontitis, and excess weight, contributing to lower circulating levels of *SOD1*.

Our hypothesis of a potential relationship between IRS-2 and SOD was demonstrated by our correlations’ findings of the gene-to-gene expression analyses, since we demonstrated strong and moderate positive correlations between *IRS2* and *SOD1* mRNA expressions in patients of T2DM_poorlyC+P, T2DM_wellC_withoutP, and Periodontitis groups. This reinforces the concept that IRS-2 could exert influence on the antioxidant system (in this case, SOD), or vice versa.

Because the role of SOD in T2DM has been extensively explored, two recent meta-analyses were developed, but the levels of this enzyme were inconclusive in the protective effect against the progression of isolated T2DM and in combination with periodontitis [49,50,51,52]. In the present study, two analyses of SOD protein in saliva were conducted: concentration (U/mL) and enzymatic activity (U/g). Here, we observed reduced SOD concentration (U/mL) in the saliva of diabetic and periodontitis patients, in agreement with the study by Karima et al. [53] that made this observation in peripheral neutrophils of patients affected by these diseases. Interestingly, for all the groups after 90 and 180 days of the NSPT, the SOD concentration (U/mL) increases in the direction of the health condition. Regarding the SOD enzymatic activity (U/g), at baseline, significantly lower levels were found in Controls in comparison with the highest SOD levels (U/g) in both T2DM+P groups (independently of metabolic control), which agrees with the study of Trivedi et al. [25]. Higher concentration of the antioxidant SOD is necessary in these diseased groups in an attempt to reach the balance of the excessive ROS triggered by the pathologies. Literature presents many more studies of SOD activity in erythrocytes than in saliva. Diabetic patients exhibit a 50% reduction in SOD activity in erythrocytes, accompanied by a significant increase in free radicals, suggesting the involvement of a common factor in tissue damage [27,52].

Regarding SOD protein and mRNA levels, our results showed high salivary SOD enzymatic activity in patients with T2DM+P. After NSPT, some groups exhibited a significant reduction in SOD1 mRNA levels in PBMCs, whereas others showed increased protein/enzymatic activity. A central explanation for these apparently divergent directions lies in the relationship between mRNA abundance and protein levels, which is well known to be complex and non-linear, varying substantially across proteins [54,55]. As discussed by Perl et al. [56], the correlation between mRNA expression and protein abundance in mammals is relatively modest, with a Pearson correlation coefficient of approximately 0.40. This discrepancy can largely be attributed to multiple layers of post-transcriptional regulation (e.g., mRNA stability, translational efficiency, protein turnover, and post-translational modifications). Therefore, Perl et al. [56] emphasize that this limited correlation complicates the direct integration of transcriptional and translational data: although mRNA expression can be informative in some contexts, it is far from a perfect predictor of protein levels.

The present study has some limitations, including challenges in selecting volunteers meeting the inclusion criteria. Additionally, rapid acquisition of certain biochemical parameters, especially HbA1c, led to the exclusion of some participants throughout the study, along with dropouts. While PBMCs provide a feasible and standardized matrix for longitudinal clinical studies assessment, it must be noted that mRNA expression in these cells serves as a systemic readout and may not fully reflect tissue-specific signaling within the periodontium. Regarding immunohistochemistry results, we faced challenges in obtaining gingival biopsy samples due to patient resistance to the recommended surgical procedure, either due to a lack of understanding of the surgery’s necessity or fears. Additionally, IRS-2 protein assessment was based on semi-quantitative immunohistochemistry in a limited subset of available gingival biopsies to verify the immunolocalization of IRS-2 in human periodontal tissue, rather than to serve as definitive quantitative protein validation of transcript-level changes. This could be considered a limitation when interpreting results; therefore, future studies enrolling a larger number of participants are needed to confirm these findings. Another limitation is the limited control of potential confounders, such as diet and medication use, which may influence systemic biochemical and molecular outcomes. In addition, age should be considered a relevant confounding factor, as the median age of the Control group was significantly lower than that of the other groups, which may have contributed to differences in systemic markers independent of periodontal status or T2DM. Moreover, this was a nonrandomized clinical study, and participants were aware of their clinical condition, although the operator who performed NSPT and clinical procedures was blinded to group allocation. Because our inclusion criteria were not intended to match groups for age, BMI, smoking history, or ethnicity, residual confounding due to baseline differences cannot be excluded and may have influenced systemic and molecular outcomes. We also acknowledge that medication use, diet, physical activity, and duration of diabetes were not controlled within the study design and are therefore considered partial limitations when interpreting the results. We recognize the need for additional studies to investigate the interaction between IRS-2 and SOD, considering a larger casuistic (although this study is sufficiently powered to reach significant differences) and diverse ethnic populations.

## 5. Conclusions

We concluded that this study provides insights into the influence of NSPT on the transcriptional and translational levels of SOD1 and IRS2 in participants affected by different combinations of T2DM and periodontitis. Moreover, moderately significant correlations were observed between IRS2 and SOD1 gene-gene expressions. These findings support further studies investigating the potential role of SOD1 and IRS2 as candidate biomarkers for T2DM and periodontitis comorbidities.

## Figures and Tables

**Table 1:** Demographic and physical characteristics of participants.

Characteristics	T2DM_poorlyC+P	T2DM_wellC+P	T2DM_without_P	Periodontitis	Control	*p*-Value
N^o^ participants	30	31	32	31	32	-
Age Median (25–75%)	53 (48–58) ^a^	58 (54–64) ^a^	59 (51–63) ^a^	53 (45–60) ^a^	37 (35–47) ^b^	0.006
Sex						
Male/Female	11/19	9/22	13/19	12/19	8/24	0.553
Ethnic group (%)						
White	19 (66.3%) ^ab^	22 (70.9%) ^ab^	24 (75.0%) ^ab^	15 (48.4%) ^b^	29 (90.6%) ^a^	0.012
Black	9 (30.0%) ^a^	7 (22.5%) ^ab^	5 (15.6%) ^ab^	12 (38.7%) ^a^	1 (3.1%) ^b^	
Pardo	2 (3.7%) ^a^	2 (6.4%) ^a^	1 (3.1%) ^a^	4 (12.9%) ^a^	2 (6.3%) ^a^	
Asian	0 (0%) ^a^	0 (0%) ^a^	2 (6.3%) ^a^	0 (0%) ^a^	0 (0%) ^a^	
Smoke (%)						
Former-smoker	10 (33.4%) ^a^	11 (35.5%) ^a^	8 (25.0%) ^a^	6 (19.4%) ^ab^	0 (0%) ^b^	0.003
Never-smoker	20 (66.6%) ^a^	20 (64.5%) ^a^	24 (75.0%) ^a^	25 (80.6%) ^ab^	32 (100%) ^b^	
BMI (Kg/m^2^)	31.3 (27.5–34.8) ^a^	29.4 (26.6–35.7) ^a^	29.7 (27.5–35.8) ^a^	29.0 (26.1–33.1) ^a^	25.3 (23.6–29.5) ^b^	0.0106
Waist-to-hip ratio (cm)	0.9 (0.9–1) ^a^	0.9 (0.9–1) ^a^	1 (0.9–1) ^a^	0.9 (0.8–1) ^a^	0.8 (0.8–0.9) ^b^	0.0389

T2DM_poorlyC+P = Type 2 Diabetes Mellitus_poorly controlled+Periodontitis; T2DM_wellC+P = Type 2 Diabetes Mellitus_well controlled+Periodontitis; T2DM_without_P = Type 2 Diabetes Mellitus without Periodontitis; % = percent; BMI = Body Mass Index. The continuous quantitative variables (Age, BMI, and Waist-to-Hip ratio) were compared among groups using the Kruskal–Wallis test ( *p* < 0.05) followed by Dunn’s post-test. The Chi-Square test was employed to assess differences among groups for nominal qualitative variables (Sex, Skin color, and Smoking).^ab^ Different letters indicate statistically significant differences among groups.

**Table 2:** Results of biochemical and periodontal parameters observed in participants at baseline, 90 days, and 180 days after NSPT.

Biochemical and Periodontal Parameter	Period	T2DM_poorlyC+P	T2DM_wellC+P	T2DM_without_P	Periodontitis	Control	*p*-Value
N° participants		30	31	32	31	32	-
Median (CI 25–75%)							
Fasting plasma glucose (mg/dL)	Baseline	157	111	135	92	92	0.0001
		(132–215) ^a^	(98.2–125.8) ^bc^	(100.3–164.3) ^ac^	(88–98) ^d^	(86.5–96) ^d^	
	90 days	171.5	114.5	140.5	95	91	0.0001
		(139.8–211.5) ^a^	(99.2–135) ^bc^	(108.8–160.8) ^ac^	(88–99.2) ^d^	(83–96.5) ^d^	
	180 days	159	121	128.5	92	91	0.0001
		(113–211) ^a^	(97–137) ^a^	(102.3–193.8) ^a^	(87–95) ^b^	(83–95) ^c^	
	Follow-Up (*p*-value)	0.6483	0.0797	0.4829	0.2583	0.4545	
HbA1c	Baseline	8.3	6.2	7.2	5.4	5.2	0.0001
		(7.4–9.9) ^a^	(5.9–6.6) ^bc^	(6.2–9) ^ac^	(5.2–5.5) ^d^	(5.1–5.4) ^d^	
	90 days	8.6	6.4	7	5.5	5.4	0.0001
		(7.5–9.7) ^a^	(5.9–6.6) ^bc^	(6.4–9.7) ^ac^	(5.2–5.6) ^d^	(5.2–5.5) ^d^	
	180 days	8.3	6.3	7.2	5.4	5.3	0.0001
		(7.7–9.3) ^a^	(5.9–6.7) ^bc^	(6.0–12.6) ^ac^	(5.2–5.6) ^d^	(5.2–5.5) ^d^	
	Follow-Up (*p*-value)	0.8061	0.1362	0.6588	0.5899	0.0468 ^$^	
Insulin (U/L)	Baseline	13.6	12.2	14.2	9.8	8.5	0.0004
		(8.2–25) ^a^	(8.1–22.1) ^a^	(9.1–21.4) ^a^	(7.3–13) ^ab^	(6.1–10.5) ^b^	
	90 days	14.4	13.2	13.1	8.6	8	0.0001
		(8.4–21.2) ^a^	(7.9–18.1) ^a^	(9.1–17.6) ^a^	(7.4–13.5) ^ab^	(5.6–8.8) ^b^	
	180 days	13.2	13.5	12.6	9.6	7.7	0.0006
		(7.3–22.1) ^a^	(8.8–16.9) ^a^	(7.6–18.8) ^a^	(6.6–13.5) ^ab^	(5.8–14.9) ^b^	
	Follow-Up (*p*-value)	0.6703	0.1856	0.8807	0.9918	0.5471	
HOMA-IR	Baseline	5	3.6	4	2.1	1.8	0.0001
		(3.4–9.6) ^a^	(2.1–6.2) ^a^	(2.9–6.8) ^a^	(1.6–3.2) ^b^	(1.3–2.4) ^b^	
	90 days	6.8	3.5	4	2.1	1.7	0.0001
		(3.3–9.2) ^a^	(2–6.3) ^bc^	(2.7–6.2) ^ac^	(1.5–3) ^bd^	(1.3–2) ^d^	
	180 days	4.9	3.8	3.9	2.1	1.7	0.0001
		(2.8–9.9) ^a^	(2.6–5.6) ^a^	(2.9–6.4) ^a^	(1.3–2.8) ^b^	(1.2–2.2) ^c^	
	Follow-Up (*p*-value)	0.9048	0.2259	0.7473	0.6692	0.5188	
Total cholesterol (mg/dL)	Baseline	186	172	187	179	188.5	0.4613
		(172.8–203.8) ^a^	(150.3–201.8) ^a^	(162–220.5) ^a^	(151–217) ^a^	(173.5–207) ^a^	
	90 days	198.5	170	198.5	189.5	165	0.3917
		(173.3–224.8) ^a^	(154–215.3) ^a^	(168.5–223) ^a^	(168.5–204.8) ^a^	(124–185) ^a^	
	180 days	184	139	168.5	125	181.5	0.0004
		(160–218) ^a^	(102–216) ^ab^	(117.3–212.3) ^ab^	(85–175) ^b^	(164.8–201.3) ^a^	
	Follow-Up (*p*-value)	0.8965	0.9025	0.6799	0.0506	0.0302 ^$^	
HDL cholesterol (mg/dL)	Baseline	46.5	51	48.5	53	61.5	0.0020
		(39–53.2) ^a^	(45.2–60) ^ab^	(40.2–58.7) ^a^	(44–66) ^ab^	(49.5–72.7) ^b^	
	90 days	48	44	41.5	46	49.5	0.0056
		(39.7–57) ^a^	(29–49) ^ab^	(28–50.5) ^ab^	(31–53) ^ab^	(40–65.5) ^b^	
	180 days	47	53	53.5	54	66.5	0.0010
		(41–54) ^a^	(41–61) ^a^	(43.2–62.7) ^ab^	(44–68) ^ab^	(52.7–74.7) ^b^	
	Follow-Up (*p*-value)	0.1933	0.7822	0.0338 ^$^	0.0693	0.4144	
LDL cholesterol (mg/dL)	Baseline	105.6	71.8	103.3	102.2	107.3	0.6619
		(34.6–127.7) ^a^	(39–91.3) ^a^	(70–133.8) ^a^	(74.4–126.2) ^a^	(83.9–131.4) ^a^	
	90 days	112.5	94.2	102.4	101.1	98.8	0.4097
		(97.8–131.3) ^a^	(76–118.9) ^a^	(75.5–130.3) ^a^	(81.2–129.3) ^a^	(81.8–120) ^a^	
	180 days	108.4	83	100.8	91	97.3	0.7384
		(85.6–125.6) ^a^	(68.2–111.8) ^a^	(64.9–136.6) ^a^	(74–130) ^a^	(75.7–111.4) ^a^	
	Follow-Up (*p*-value)	0.0239 ^#^	0.0939	0.9922	0.5435	0.2163	
Triglycerides (mg/dL)	Baseline	159.5	150.5	169	128	92.5	0.0011
		(110.5–232.3) ^a^	(103.5–195.3) ^ab^	(108–199) ^ab^	(89–201) ^abc^	(59–148) ^c^	
	90 days	163.5	154.5	159	118.5	89	0.0001
		(122–233) ^a^	(106–199) ^a^	(96.5–242) ^a^	(81–182.3) ^a^	(53.2–132.3) ^b^	
	180 days	148	139	168.5	125	83	0.0001
		(96–196) ^a^	(102–216) ^a^	(117.3–212.3) ^a^	(85–175) ^ab^	(68.5–150.5) ^b^	
	Follow-Up (*p*-value)	0.9999	0.9447	0.6625	0.4545	0.4772	
vLDL	Baseline	31.9	30.1	33.8	25.6	18.5	0.0011
		(22.1–46.4) ^a^	(20.7–39.8) ^ab^	(21.6–39.8) ^a^	(17.8–40.2) ^ab^	(11.8–29.6) ^b^	
	90 days	32.7	30.9	31.8	23.7	17.8	0.0001
		(24.4–46.6) ^a^	(21.2–39.8) ^a^	(19.3–48.4) ^a^	(16.2–36.4) ^a^	(10.6–26.4) ^b^	
	180 days	29.6	27.8	33.7	25	16.6	0.0001
		(19.2–39.2) ^a^	(20.4–43.2) ^a^	(23.4–42.4) ^a^	(17–35) ^ab^	(13.7–30.1) ^b^	
	Follow-Up (*p*-value)	0.9999	0.9447	0.7834	0.4545	0.4772	
Non-HDL-C	Baseline	137	125	133.5	98	130.5	0.4288
		(116.8–161.3) ^a^	(101.5–142) ^a^	(101–172.5) ^a^	(127–159) ^a^	(102–151.8) ^a^	
	90 days	150.5	117	142	131	115.5	0.0582
		(117–169.3) ^a^	(104.5–164.8) ^a^	(107–169.5) ^a^	(112.5–155.3) ^a^	(97.7–141) ^a^	
	180 days	138	121	137	118	113.5	0.1876
		(104–167) ^a^	(97–159) ^a^	(99.7–181) ^a^	(103–148) ^a^	(96–136.8) ^a^	
	Follow-Up (*p*-value)	0.2043	0.4053	0.6597	0.0778	0.0123 *	
N° teeth	Baseline	24	23	23	26	27	0.0019
		(21–26) ^a^	(21–26) ^a^	(20–28) ^a^	(23–28) ^a^	(24–28) ^ab^	
	90 days	24	23	23	25	27	0.0007
		(21–26) ^a^	(21–26) ^a^	(20–28) ^a^	(23–28) ^a^	(24–28) ^ab^	
	180 days	24	23	23	25	27	0.0005
		(21–26) ^a^	(21–25) ^a^	(20–27) ^a^	(23–27) ^ab^	(24–28) ^b^	
	Follow-Up (*p*-value)	0.9324	0.9267	0.3679	0.8834	0.6065	
Bleeding on probing	Baseline	53	56.4	28.7	50.6	25	0.0001
		(47.7–64) ^a^	(41.9–61.6) ^a^	(18–39.1) ^b^	(38.2–60.6) ^a^	(14–33) ^b^	
	90 days	44	42.6	21.4	33.3	21.7	0.0001
		(33.1–62.5) ^a^	(33.5–49) ^a^	(14–30.2) ^b^	(20.2–43.3) ^ab^	(17.6–31.8) ^b^	
	180 days	42.91	38.93	21.69	35.33	19.05	<0.0001
		(28.07–48.94) ^a^	(29.14–50.98) ^a^	(14.91–27.06) ^b^	(21.05–43.83) ^a^	(12.05–24.65) ^b^	
	Follow-Up (*p*-value)	0.0004 *	<0.0001 *^$^	0.0064 *	<0.0001 *^$^	0.0067 *^#^	
Marginal bleeding index	Baseline	26.9	20.7	4.6	11.5	2.95	0.0001
		(39.4–10.2) ^a^	(35.7–12.5) ^a^	(8.7–2.9) ^bc^	(28.4–6.3) ^ac^	(5.3–1.8) ^b^	
	90 days	15.1	9.2	3.2	7.1	3.6	0.0001
		(8.2–25.2) ^a^	(7.3–14.2) ^a^	(1–9.1) ^bc^	(4.2–13.2) ^ac^	(0.9–5.4) ^b^	
	180 days	13.85	12.25	12.55	1.7	1	<0.0001
		(3.42–28.90) ^a^	(3.72–23.70) ^a^	(5.22–23.40) ^a^	(0.00–4) ^b^	(0.0–3.10) ^b^	
	Follow-Up (*p*-value)	0.0020 *^$^	0.0085 ^$^	0.0030 *	0.0016 *	0.0022 *^#^	
Plaque index	Baseline	46.5	46.2	29.4	32.1	18.7	0.0001
		(30.3–65.2) ^a^	(26.8–56.5) ^a^	(21.6–45.3) ^ac^	(22–48.2) ^a^	(11.4–30.5) ^bc^	
	90 days	29.4	27.7	19.4	22.3	14.1	0.0001
		(20–42) ^a^	(19.2–39.8) ^ab^	(12.6–32.3) ^bc^	(14.3–34.8) ^ac^	(10.3–23.3) ^c^	
	180 days	30.35	28.70	17.05	202.20	10.70	<0.0001
		(20.63–44.08) ^a^	(16.10–34.90) ^a^	(9.85–22.65) ^bc^	(14–36.8) ^ab^	(5.45–20.05) ^c^	
	Follow-Up (*p*-value)	0.0006 *^$^	0.0004 *^$^	<0.0001 *^$^	0.0027 *^$^	0.0001 *	
PD ≤ 3 mm	Baseline	74	71.5	98	73	98	0.0001
		(62.2–82) ^a^	(61.2–78) ^a^	(96.2–98.7) ^bc^	(56–84) ^ac^	(98–99) ^b^	
	90 days	82	81.5	99	83	99	0.0001
		(64–86) ^a^	(67–88.5) ^a^	(98–100) ^b^	(72–92) ^a^	(98–100) ^b^	
	180 days	85.5	86	99.5	87	99	<0.0001
		(78.5–92) ^a^	(71.25–91.75) ^a^	(99–100) ^b^	(73–94) ^a^	(99–100) ^b^	
	Follow-Up (*p*-value)	0.0004 *	<0.0001 *^$^	<0.0001 *^$^	<0.0001 *^$^	0.0051 *	
PD 4–5 mm	Baseline	21	21	2	21	2	0.0001
		(16.5–33.2) ^a^	(16–26) ^a^	(1.2–3) ^b^	(14–29) ^a^	(1–2) ^b^	
	90 days	12.7	10	0	8	0	0.0001
		(12.7–32.2) ^a^	(10–26.2) ^a^	(0–2) ^b^	(8–22) ^a^	(0–2) ^b^	
	180 days	13	12.50	0.5	11	1	<0.0001
		(6.25–17.50) ^a^	(8–22.5) ^a^	(0–1) ^b^	(5–17) ^a^	(0–1) ^b^	
	Follow-Up (*p*-value)	0.0003 *	0.0023 *	<0.0001 *	<0.0001 *^$^	0.0012 *	
PD ≥ 6 mm	Baseline	4	5.5	0	7	0	0.0001
		(1–8) ^a^	(1.2–12) ^a^	(0–0) ^b^	(2–14) ^a^	(0–0) ^b^	
	90 days	2	3.5	0	2	0	0.0001
		(0–7.25) ^a^	(1–8.2) ^a^	(0–0) ^b^	(0–8) ^a^	(0–0) ^b^	
	180 days	1	1	0	2	0	<0.0001
		(0–4) ^a^	(0–6.75) ^a^	(0–0) ^b^	(0–7) ^a^	(0–0) ^b^	
	Follow-Up (*p*-value)	0.0006 *	<0.0001 *^$^	0.050	<0.0001 *^$^	0.7788	
CAL 2 mm (% of the sites)	Baseline	37	32	86.5	43	89.5	0.0001
		(21.1–47) ^a^	(25–41.7) ^a^	(81–92) ^b^	(26–81) ^a^	(83–94) ^b^	
	90 days	41.5	40.5	89	43	91.5	0.0001
		(21–51.5) ^a^	(31.5–55) ^a^	(86–95) ^b^	(32–59) ^a^	(83.7–96) ^b^	
	180 days	49	41	90	49	92	<0.0001
		(40–56.75) ^a^	(27.75–59.75) ^a^	(83.25–95) ^b^	(31–60) ^ac^	(86.5–96.75) ^bc^	
	Follow-Up (*p*-value)	0.0018 *	0.0005 *^$^	0.1613	0.0615	0.0195 *	
CAL 3–4 mm (% of the sites)	Baseline	42.5	44	12.5	40	10.5	0.0001
		(36–52.2) ^a^	(36.5–51.7) ^a^	(7–16.7) ^b^	(19–47) ^a^	(6–15.7) ^b^	
	90 days	47	41.5	10	39	8.5	0.0001
		(36.7–54.2) ^a^	(33.2–47.7) ^a^	(5–12.7) ^b^	(34–46) ^a^	(4–14.7) ^b^	
	180 days	44	46	9	37	8	<0.0001
		(33–47.75) ^a^	(36–52) ^a^	(4.25–13.75) ^b^	(29–47) ^a^	(3.25–12) ^b^	
	Follow-Up (*p*-value)	0.5241	0.1364	0.5034	0.7919	0.0311 *	
CAL ≥ 5 mm (% of the sites)	Baseline	17	19	1	11	0	0.0001
		(8–28.5) ^a^	(9–27.2) ^a^	(0–2.7) ^b^	(1–27) ^a^	(0–0) ^b^	
	90 days	12	13.5	0.5	15	0	0.0001
		(6–19.7) ^a^	(5.5–22) ^a^	(0–2) ^b^	(6–24) ^a^	(0–0.7) ^b^	
	180 days	8	9.5	0	15	0	<0.0001
		(3.5–22) ^a^	(4–24) ^a^	(0–2) ^b^	(3–21) ^a^	(0–1) ^b^	
	Follow-Up (*p*-value)	<0.0001 *^$^	<0.0001 *^$^	0.0587	0.1342	0.8717	

T2DM_poorlyC+P = Type 2 Diabetes Mellitus_poorly controlled+Periodontitis; T2DM_wellC+P = Type 2 Diabetes Mellitus_well controlled+Periodontitis; T2DM_without_P = Type 2 Diabetes Mellitus without Periodontitis; CI = confidence interval; PD = Probing Depth; CAL = Clinical Attachment Loss. The Kruskal–Wallis test (*p* < 0.05) was performed, followed by Dunn’s post-test. Different letters ^abcd^ mean significant differences among groups. For the comparison between baseline, 90, and 180 days, the Friedman’s test was performed. Follow-up (*p*-value): * = baseline ≠ 180 days; # = 90 days ≠ 180 days; $ = baseline ≠ 90 days.

**Table 3:** Salivary SOD protein levels at baseline, 90 days, and 180 days after NSPT.

Period	T2DM_poorlyC+P	T2DM_wellC+P	T2DM_poorlyC_without_P	T2DM_wellC_without_P	Periodontitis	Control
SOD concentration (U/mL)						
Baseline	1.23 ± 0.34 ^a,b^	1.15 ± 0.33 ^a^	1.43 ± 0.28 ^b,^*	1.43 ± 0.27 ^a,b^	1.39 ± 0.29 ^a,b^	1.42 ± 0.28 ^b,^*
90 days	1.54 ± 0.35 ^a^	1.43 ± 0.34 ^a^	1.46 ± 0.31 ^a#^	1.42 ± 0.46 ^a,#^	1.49 ± 0.28 ^a^	1.32 ± 0.26 ^a,#^
180 days	1.62 ± 0.70 ^a^	1.61 ± 0.56 ^a^	2.12 ± 0.76 ^a,^*^,#^	2.28 ± 1.29 ^a,#^	1.74 ± 0.67 ^a^	1.95 ± 0.80 ^a,^*^,#^
SOD/Total Protein activity (U/g)						
Baseline	1.33 (0.25–2.12) ^a^	1.02 (0.16–1.74) ^a,b^	0.23 (0.19–0.27) ^a,b,$,^*	0.24 (0.19–0.26) ^a,b,$,^*	0.70 (0.20–2.43) ^a^	0.22 (0.18–0.26) ^b,$,^*
90 days	0.97 (0.73–1.48)	1.15 (0.82–1.77)	1.68 (0.98–2.02) ^$^	1.69 (0.972–2.15) ^$^	1.04 (0.75–1.99)	1.14 (0.71–1.95) ^$^
180 days	1.09 (0.62–2.04) ^a,b^	0.82 (0.68–1.15) ^b^	1.47 (1.25–1.89) ^a,^*	1.60 (1.18–2.13) ^a,^*	1.86 (1.05–2.31) ^a^	1.75 (1.37–2.14) ^a,^*

T2DM_poorlyC+P = Type 2 Diabetes Mellitus_poorly controlled+Periodontitis; T2DM_wellC+P = Type 2 Diabetes Mellitus_well controlled+Periodontitis; T2DM_poorlyC_without_P = Type 2 Type 2 Diabetes Mellitus_poorly controlled_without Periodontitis; T2DM_wellC_without_P = Type 2 Type 2 Diabetes Mellitus_well controlled_without Periodontitis. The groups at baseline, 90, and 180 days were compared using the one-way ANOVA test, followed by Tukey’s post-test (SOD (U/mL). The Kruskal–Wallis test was performed, followed by Dunn’s post-test. Different letters (SOD/Total Protein (U/g)); ^ab^ mean significant differences among groups. Friedman’s non-parametric test was used, followed by Dunn’s post-test for all longitudinal evaluations. Follow-up (*p*-value): * = baseline ≠ 180 days; # = 90 days ≠ 180 days; $ = baseline ≠ 90 days.

## Data Availability

Written informed consent has been obtained from the patients to publish this paper.

## Supplementary Materials

The following supporting information can be downloaded at: https://www.mdpi.com/article/10.3390/biomedicines14040742/s1, Table S1: Genes investigated and TaqMan assay codes used in qPCR reactions to assess gene expression in patients; Figure S1: Correlation between SOD1 and IRS2 mRNA levels, SOD enzyme activity, demographic, physical, biochemical, and periodontal parameters (Group: T2DM_poorlyC+P-Baseline); Figure S2: Correlation between SOD1 and IRS2 mRNA levels, SOD enzyme activity, demographic, physical, biochemical, and periodontal parameters (Group: T2DM_wellC+P-Baseline); Figure S3: Correlation between SOD1 and IRS2 mRNA levels, SOD enzyme activity, demographic, physical, biochemical, and periodontal parameters (Group: T2DM_without_P-Baseline); Figure S4: Correlation between SOD1 and IRS2 mRNA levels, SOD enzyme activity, demographic, physical, biochemical, and periodontal parameters (Group: Periodontitis-Baseline); Figure S5:Correlation between SOD1 and IRS2 mRNA levels, SOD enzyme activity, demographic, physical, biochemical, and periodontal parameters (Group: Control-Baseline); Figure S6: Correlation between SOD1 and IRS2 mRNA levels, SOD enzyme activity, demographic, physical, biochemical, and periodontal parameters (Group: T2DM_poorlyC+P- 90 days); Figure S7: Correlation between SOD1 and IRS2 mRNA levels, SOD enzyme activity, demographic, physical, biochemical, and periodontal parameters (Group: T2DM_wellC+P-90 days); Figure S8: Correlation between SOD1 and IRS2 mRNA levels, SOD enzyme activity, demographic, physical, biochemical, and periodontal parameters (Group: T2DM_without_P-90 days); Figure S9: Correlation between SOD1 and IRS2 mRNA levels, SOD enzyme activity, demographic, physical, biochemical, and periodontal parameters (Group: Periodontitis- 90 days); Figure S10: Correlation between SOD1 and IRS2 mRNA levels, SOD enzyme activity, demographic, physical, biochemical, and periodontal parameters (Group:Control-90 days); Figure S11: Correlation between SOD1 and IRS2 mRNA levels, SOD enzyme activity, demographic, physical, biochemical, and periodontal parameters (Group:T2DM_poorlyC+P-180 days); Figure S12: Correlation between SOD1 and IRS2 mRNA levels, SOD enzyme activity, demographic, physical, biochemical, and periodontal parameters (Group: T2DM_wellC+P- 180days); Figure S13: Correlation between SOD1 and IRS2 mRNA levels, SOD enzyme activity, demographic, physical, biochemical, and periodontal parameters (Group: T2DM_without_P- 180 days); Figure S14: Correlation between SOD1 and IRS2 mRNA levels, SOD enzyme activity, demographic, physical, biochemical, and periodontal parameters (Group: Periodontitis- 180 days); Figure S15: Correlation between SOD1 and IRS2 mRNA levels, SOD enzyme activity, demographic, physical, biochemical, and periodontal parameters (Group: Control- 180 days).

## Abbreviations

The following abbreviations are used in this manuscript:

NSPT	Non-surgical periodontal therapy
SOD	Superoxide Dismutase
SOD1	Superoxide Dismutase 1
IRS2	Insulin Receptor Substrate type 2
IRS1	Insulin Receptor Substrate type 1
T2DM	Type 2 Diabetes Mellitus
mRNA	Messenger RNA
HbA1c	Glycated hemoglobin
PI3K-PKB	Phosphatidylinositol 3-kinase and protein kinase B
JNK-AP-1	c-Jun N-terminal kinase-Activator Protein-1
IKK-NF-kB	I kappa B kinase- Nuclear factor kappa-B
GLUT4	Glucose transporter type 4
Nrf2	NF-E2-related factor 2
Keap1	Kelch-like ECH-associated protein
TC	Total Cholesterol
O_2_•−	Superoxide radical anion
H_2_O_2_	Hydrogen peroxide
GPx	Glutathione peroxidase
ROS	Reactive oxygen species
TNF-α	Tumor necrosis factor-alpha
PBMCs	Peripheral blood mononuclear cells
TREND	Transparent Reporting of Evaluations with Nonrandomized Designs
ReBEC	Brazilian Clinical Trials Registry
HOMA	Homeostasis model assessment
IR	Insulin resistance
HDL-Cholesterol	High-density lipoprotein Cholesterol
LDL-Cholesterol	low-density lipoprotein cholesterol
vLDL	very-low-density lipoprotein
CVD	cardiovascular disease
VPI	Visible Plaque Index
MBI	Marginal Bleeding Index
BoP	Bleeding on Probing
PD	Probing Depth
CAL	Clinical Attachment Loss
XOD	Xanthine-xanthine oxidase
BSA	Bovine serum albumin
I.N.T.	2-(4-iodophenyl)-3-(4-nitrophenol)-5-phenyltetrazolium chloride
