# Supplementary material for: Longitudinal Study of the Influence of Periodontal Treatment on the Levels of Insulin Receptor Substrate-2 and Superoxide Dismutase 1 in Individuals with Type 2 Diabetes Mellitus
Source: Biomedicines. 2026 Mar 24;14(4):742. doi: 10.3390/biomedicines14040742 (PMC13113949; doi:10.3390/biomedicines14040742)
Supplement: Supplementary file 1 [file biomedicines-14-00742-s001.zip › biomedicines-4156027-supplementary.pdf]

Supplementary Material

Longitudinal study of the influence of periodontal treatment on the levels of Insulin receptor substrate-2 and Superoxide Dis-mutase 1 in individuals with type 2 diabetes mellitus

Materials and Methods

Sample size calculation and researcher calibration

The reproducibility among two clinicians (IGN) and (MGGM) of the periodontal exams was evaluated in seven individuals with generalized periodontitis, stages III and IV, grades B and C, with a minimum of 15 teeth in the oral cavity. The parameter used for calibration was periodontal probing depth (PD), and the comprehensive periodontal exams were conducted on the same day, with an interval of at least 1 hour between them. Periodontal exam reproducibility was evaluated using the Intraclass Correlation Coefficient, which ranged from 0.90 to 0.93 for PD and from 0.91 to 0.94 for clinical attachment loss (CAL).

Study Population

The diagnosis of T2DM was made by an endocrinologist who monitored the glycemic levels of each participant by evaluation of glycated hemoglobin (HbA1c) values, according to the American Academy of Diabetes (American Diabetes Association Professional Practice, 2022)., which classify individuals affected by T2DM as: normoglycemic (HbA1c ≤ 5.6%); well controlled (wellC) T2DM (HbA1c 6.5% -7%); or poorly controlled (poorlyC) T2DM (HbA1c ≥ 7.1%).

Periodontal Evaluations

During the active phase of NSPT, sessions of Scaling and Root Planning (SRP) were performed on the entire dental arch, using manual curettes, in one quadrant or sextant per week (depending on the extent and severity of periodontitis), with each session lasting 45 to 60 minutes. All patients received prophylaxis with a rubber cup and prophylactic paste, as well as individualized oral hygiene instructions.

The active phase of NSPT lasted 4 to 8 weeks, depending on the severity of the case. All individuals were reassessed 45 days after the completion of active periodontal therapy. After the completion of active periodontal treatment, maintenance appointments were conducted every 15 days for a period of 45 days, completing a follow up of 90 days. Following the 90-day reassessment, all patients received periodontal maintenance every 30 days until the completion of the 180-day study period.

After the completion of active periodontal treatment, reassessments were conducted between 90 and 180 days. During these reassessment periods, if the presence of residual pockets with BoP ≥6 mm was identified [1,2], all participants with

this condition for therapeutic decision-making: systemic health status of patients, oral hygiene conditions (VPI <20% or improvement in visible plaque index of at least 30% compared to baseline); residual PD (sites with residual PD ≥6mm with BoP); extent of marginal inflammation (MBI in <20% of sites or improvement of at least 30% compared to baseline); presence of root sensitivity; location of the site; and the condition of adjacent periodontal sites.

Results

Following, Supplementary Figures 1 -15 presents correlation findings obtained from each group at each study period among SOD1 and IRS2 mRNA levels, SOD enzyme activity, demographic, physical, biochemical, and periodontal parameters.

References

1. Sanz, M.; Herrera, D.; Kebschull, M.; Chapple, I.; Jepsen, S.; Beglundh, T.; et al. Treatment of Stage I–III Periodontitis: The EFP S3 Level Clinical Practice Guideline. *J. Clin. Periodontol.* **2020**, *47*(Suppl 22), 4–60.
2. Sanz-Sanchez, I.; Montero, E.; Citterio, F.; Romano, F.; Molina, A.; Aimetti, M. Efficacy of Access Flap Procedures Compared to Subgingival Debridement in the Treatment of Periodontitis: A Systematic Review and Meta-Analysis. *J. Clin. Periodontol.* **2020**, *47*(Suppl 22), 282–302.

Table S1 - Genes investigated and TaqMan assay codes used in qPCR reactions to assess gene expression in patients

Gene	Codes	Acession	(exons)	Amplicon (bp)
IRS2	Hs00275843_s1	NM_003749.2	1-1	70
SOD1	Hs00533490_m1	NM_000454.4	1-2	60
GAPDH	Hs02758991_g1	NM_002046.4	7-8	93
ACTG	Hs03044422_g1	NM_001199954.1	5 - 6	132

Supplementary Figure S1- Correlation between SOD1 and IRS2 mRNA levels, SOD enzyme activity, demographic, physical, biochemical, and periodontal parameters (Group: T2DM_poorlyC+P-Baseline).

**T2DM_poorlyC+P Baseline**	*SOD1*	*IRS2*	SOD (U/g)	Fasting glucose	HbA1c (%)	Insulin (U/L)	HOMA	BMI	Waist-hip ratio	Triglycerides	Total cholesterol	HDL cholesterol	LDL cholesterol	VLDL	Non-HDL-C	BoP(%)	PD 4-5 mm	PD ≥6 mm	CAL 3-4 mm	CAL≥ 5mm
SOD1		0.285	0.146	-0.032	0.215	-0.491	-0.496	-0.416	-0.265	-0.107	-0.070	0.159	0.128	-0.107	-0.032	-0.079	-0.069	-0.197	-0.004	-0.016
IRS2	0.285		0.105	-0.192	0.154	-0.254	-0.291	-0.166	0.027	-0.169	-0.093	0.020	0.044	-0.169	-0.072	0.191	0.144	-0.182	0.213	-0.072
SOD (U/g)	0.146	0.105		0.070	-0.245	-0.384	-0.370	-0.388	-0.049	-0.149	0.056	0.079	0.062	-0.149	-0.036	-0.090	0.195	-0.359	-0.190	-0.052
Fasting glucose	-0.032	-0.192	0.070		-0.020	-0.064	0.257	-0.287	0.108	0.180	0.083	-0.227	0.035	0.180	0.140	-0.188	-0.298	-0.038	-0.340	0.031
HbA1c (%)	0.215	0.154	-0.245	-0.020		0.029	0.057	0.086	-0.058	-0.159	-0.073	0.228	-0.028	-0.159	-0.041	0.044	-0.248	-0.006	-0.078	0.069
Insulin (U/L)	-0.491	-0.254	-0.384	-0.064	0.029		0.924	0.364	0.416	0.282	-0.200	-0.153	-0.314	0.282	-0.187	0.094	0.243	0.440	0.157	0.337
HOMA	-0.496	-0.291	-0.370	0.257	0.057	0.924		0.262	0.467	0.415	-0.155	-0.243	-0.338	0.415	-0.113	0.017	0.108	0.370	0.068	0.346
BMI	-0.416	-0.166	-0.388	-0.287	0.086	0.364	0.262		0.092	0.141	0.104	0.139	-0.112	0.141	0.028	-0.153	-0.154	0.044	0.290	-0.182
Waist-hip ratio	-0.265	0.027	-0.049	0.108	-0.058	0.416	0.467	0.092		0.313	-0.198	-0.457	-0.379	0.313	-0.128	0.084	0.149	0.174	0.175	0.071
Triglycerides	-0.107	-0.169	-0.149	0.180	-0.159	0.282	0.415	0.141	0.313		0.305	-0.204	-0.135	1.000	0.309	-0.198	-0.023	0.080	-0.048	0.052
Total cholesterol	-0.070	-0.093	0.056	0.083	-0.073	-0.200	-0.155	0.104	-0.198	0.305		0.428	0.738	0.305	0.906	-0.035	-0.255	-0.267	-0.159	-0.347
HDL cholesterol	0.159	0.020	0.079	-0.227	0.228	-0.153	-0.243	0.139	-0.457	-0.204	0.428		0.222	-0.204	0.125	0.166	-0.069	-0.145	-0.002	-0.083
LDL cholesterol	0.128	0.044	0.062	0.035	-0.028	-0.314	-0.338	-0.112	-0.379	-0.135	0.738	0.222		-0.135	0.840	0.034	-0.172	-0.196	-0.185	-0.243
VLDL	-0.107	-0.169	-0.149	0.180	-0.159	0.282	0.415	0.141	0.313	1.000	0.305	-0.204	-0.135		0.309	-0.198	-0.023	0.080	-0.048	0.052
Non-HDL-C	-0.032	-0.072	-0.036	0.140	-0.041	-0.187	-0.113	0.028	-0.128	0.309	0.906	0.125	0.840	0.309		-0.054	-0.269	-0.183	-0.162	-0.291
BoP(%)	-0.079	0.191	-0.090	-0.188	0.044	0.094	0.017	-0.153	0.084	-0.198	-0.035	0.166	0.034	-0.198	-0.054		0.563	0.347	0.054	0.343
PD 4-5 mm	-0.069	0.144	0.195	-0.298	-0.248	0.243	0.108	-0.154	0.149	-0.023	-0.255	-0.069	-0.172	-0.023	-0.269	0.563		0.555	0.103	0.647
PD ≥6 mm	-0.197	-0.182	-0.359	-0.038	-0.006	0.440	0.370	0.044	0.174	0.080	-0.267	-0.145	-0.196	0.080	-0.183	0.347	0.555		-0.125	0.769
CAL 3-4 mm	-0.004	0.213	-0.190	-0.340	-0.078	0.157	0.068	0.290	0.175	-0.048	-0.159	-0.002	-0.185	-0.048	-0.162	0.054	0.103	-0.125		-0.181
CAL≥ 5mm	-0.016	-0.072	-0.052	0.031	0.069	0.337	0.346	-0.182	0.071	0.052	-0.347	-0.083	-0.243	0.052	-0.291	0.343	0.647	0.769	-0.181	1.000

T2DM_poorlyC+P= type 2 Diabetes Mellitus poorly Controlled+ Periodontitis; *SOD1*= Superoxide dismutase 1; *IRS2*= Insulin Receptor Substrate 2; HbA1c= Glycated hemoglobin; HOMA= Homeostasis model assessment; BMI= Body mass index; VLDL= Very low-density lipoprotein; BoP= Bleeding on probing; PD= Probing depth; CAL= Clinical attachment level. Values above light gray meant p values. The green colors meant that there was a positive significant correlation, and the red color meant negative significant correlation. Upper diagonal= p values. Lower diagonal values= p= Spearman's correlation coefficients.

Supplementary Figure S2- Correlation between SOD1 and IRS2 mRNA levels, SOD enzyme activity, demographic, physical, biochemical, and periodontal parameters (Group: T2DM_wellC+P-Baseline).

**T2DM_wellC+P Baseline**	*SOD1*	*IRS2*	SOD (U/g)	Fasting glucose	HbA1c (%)	Insulin (U/L)	HOMA	BMI	Waist-hip ratio	Triglycerides	Total cholesterol	HDL cholesterol	LDL cholesterol	VLDL	Non-HDL-C	BoP(%)	PD 4-5 mm	PD ≥6 mm	CAL 3-4 mm	CAL≥ 5mm
SOD1		0.407	0.221	0.309	-0.056	-0.120	-0.049	-0.064	-0.091	0.144	-0.101	-0.210	-0.113	0.144	-0.076	-0.118	0.203	0.033	0.148	0.112
IRS2	0.407		0.276	0.277	0.103	-0.188	-0.078	-0.290	0.044	0.272	0.090	-0.277	0.055	0.272	0.147	0.028	-0.147	0.003	0.193	0.297
SOD (U/g)	0.2207	0.2764		0.0409	-0.0228	-0.2773	-0.2190	-0.1044	-0.1826	-0.0200	0.0458	0.0942	-0.0069	-0.0200	-0.0247	-0.0244	0.1149	0.1891	-0.2510	0.1773
Fasting glucose	0.309	0.277	0.041		0.701	0.318	0.547	0.165	0.276	0.507	0.037	-0.176	-0.032	0.507	0.082	-0.085	0.128	0.130	0.140	0.242
HbA1c (%)	-0.056	0.103	-0.023	0.701		0.310	0.482	0.233	0.234	0.234	0.084	-0.070	0.056	0.234	0.065	0.093	0.198	0.175	0.069	0.281
Insulin (U/L)	-0.120	-0.188	-0.277	0.318	0.310		0.949	0.322	0.328	0.651	-0.005	-0.398	-0.117	0.651	0.100	-0.016	-0.197	-0.071	0.028	0.030
HOMA	-0.049	-0.078	-0.219	0.547	0.482	0.949		0.339	0.411	0.698	-0.016	-0.412	-0.145	0.698	0.095	-0.010	-0.112	-0.057	0.106	0.094
BMI	-0.064	-0.290	-0.104	0.165	0.233	0.322	0.339		0.073	-0.029	-0.181	-0.157	-0.113	-0.029	-0.123	-0.112	0.006	0.110	0.018	-0.177
Waist-hip ratio	-0.091	0.044	-0.183	0.276	0.234	0.328	0.411	0.073		0.379	-0.117	-0.434	-0.148	0.379	0.028	-0.020	-0.134	-0.237	0.370	-0.137
Triglycerides	0.144	0.272	-0.020	0.507	0.234	0.651	0.698	-0.029	0.379		0.130	-0.516	-0.061	1.000	0.270	0.091	-0.081	0.008	0.101	0.183
Total cholesterol	-0.101	0.090	0.046	0.037	0.084	-0.005	-0.016	-0.181	-0.117	0.130		0.430	0.959	0.130	0.964	0.290	0.107	0.104	-0.103	0.028
HDL cholesterol	-0.210	-0.277	0.094	-0.176	-0.070	-0.398	-0.412	-0.157	-0.434	-0.516	0.430		0.453	-0.516	0.226	0.171	0.400	0.195	-0.190	0.002
LDL cholesterol	-0.113	0.055	-0.007	-0.032	0.056	-0.117	-0.145	-0.113	-0.148	-0.061	0.959	0.453		-0.061	0.918	0.247	0.100	0.103	-0.067	-0.001
VLDL	0.144	0.272	-0.020	0.507	0.234	0.651	0.698	-0.029	0.379	1.000	0.130	-0.516	-0.061		0.270	0.091	-0.081	0.008	0.101	0.183
Non-HDL-C	-0.076	0.147	-0.025	0.082	0.065	0.100	0.095	-0.123	0.028	0.270	0.964	0.226	0.918	0.270		0.242	-0.001	-0.010	-0.004	-0.035
BoP(%)	-0.118	0.028	-0.024	-0.085	0.093	-0.016	-0.010	-0.112	-0.020	0.091	0.290	0.171	0.247	0.091	0.242		0.525	0.266	0.117	0.339
PD 4-5 mm	0.203	-0.147	0.115	0.128	0.198	-0.197	-0.112	0.006	-0.134	-0.081	0.107	0.400	0.100	-0.081	-0.001	0.525		0.297	0.136	0.386
PD ≥6 mm	0.033	0.003	0.189	0.130	0.175	-0.071	-0.057	0.110	-0.237	0.008	0.104	0.195	0.103	0.008	-0.010	0.266	0.297		-0.478	0.631
CAL 3-4 mm	0.148	0.193	-0.251	0.140	0.069	0.028	0.106	0.018	0.370	0.101	-0.103	-0.190	-0.067	0.101	-0.004	0.117	0.136	-0.478		-0.176
CAL≥ 5mm	0.112	0.297	0.177	0.242	0.281	0.030	0.094	-0.177	-0.137	0.183	0.028	0.002	-0.001	0.183	-0.035	0.339	0.386	0.631	-0.176	

T2DM_wellC+P= type 2 Diabetes Mellitus well Controlled + Periodontitis; *SOD1*= Superoxide dismutase 1; *IRS2*= Insulin Receptor Substrate 2; HbA1c= Glycated hemoglobin; HOMA= Homeostasis model assessment; BMI= Body mass index; VLDL= Very low-density lipoprotein; BoP= Bleeding on probing; PD= Probing depth; CAL= Clinical attachment level. Values above light gray meant p values. The green colors meant that there was a positive significant correlation, and the red color meant negative significant correlation. Upper diagonal= p values. Lower diagonal values= p= Spearman's correlation coefficients.

Supplementary Figure S3- Correlation between SOD1 and IRS2 mRNA levels, SOD enzyme activity, demographic, physical, biochemical, and periodontal parameters (Group: T2DM_without_P-Baseline).

**T2DM_without_P Baseline**	*SOD1*	*IRS2*	*SOD (U/g)*	Fasting glucose	HbA1c (%)	Insulin (U/L)	HOMA	BMI	Waist-hip ratio	Triglycerides	Total cholesterol	HDL cholesterol	LDL cholesterol	VLDL	Non-HDL-C	BoP(%)	PD 4-5 mm	PD ≥6 mm	CAL 3-4 mm	CAL≥ 5mm
SOD1		0.367	0.170	0.004	-0.164	-0.265	-0.277	-0.101	-0.123	-0.227	0.054	0.379	-0.118	-0.229	-0.087	0.123	0.058	0.113	-0.124	-0.059
IRS2	0.367		0.316	0.218	0.062	-0.233	-0.092	-0.180	0.015	-0.086	-0.084	0.132	-0.228	-0.020	-0.155	-0.219	0.165	-0.143	-0.007	0.032
SOD (U/g)	0.170	0.316		0.137	0.096	-0.165	-0.064	-0.331	0.096	0.063	0.127	-0.057	0.002	0.093	0.072	0.121	0.123	-0.329	-0.055	-0.189
Fasting glucose	0.004	0.218	0.137		0.537	-0.065	0.334	-0.118	0.232	0.552	0.165	-0.421	-0.107	0.585	0.288	0.307	0.145	0.256	0.130	-0.001
HbA1c (%)	-0.164	0.062	0.096	0.537		0.229	0.431	-0.171	0.161	0.239	0.105	-0.233	-0.024	0.289	0.179	-0.003	0.286	0.195	0.028	0.064
Insulin (U/L)	-0.265	-0.233	-0.165	-0.065	0.229		0.886	0.468	0.160	0.225	-0.085	-0.283	-0.197	0.212	-0.057	-0.106	0.222	0.420	0.161	0.130
HOMA	-0.277	-0.092	-0.064	0.334	0.431	0.886		0.377	0.369	0.446	0.013	-0.450	-0.224	0.458	0.102	-0.006	0.264	0.440	0.241	0.195
BMI	-0.101	-0.180	-0.331	-0.118	-0.171	0.468	0.377		-0.083	0.167	0.001	-0.116	-0.002	0.091	0.029	-0.072	0.063	0.246	0.235	-0.136
Waist-hip ratio	-0.123	0.015	0.096	0.232	0.161	0.160	0.369	-0.083		0.078	-0.185	-0.264	-0.198	0.109	-0.083	-0.025	-0.149	0.133	0.212	0.243
Triglycerides	-0.227	-0.086	0.063	0.552	0.239	0.225	0.446	0.167	0.078		0.407	-0.577	0.073	1.000	0.585	0.250	0.254	0.247	-0.080	0.148
Total cholesterol	0.054	-0.084	0.127	0.165	0.105	-0.085	0.013	0.001	-0.185	0.407		0.099	0.753	0.340	0.935	-0.152	0.089	0.118	0.157	0.150
HDL cholesterol	0.379	0.132	-0.057	-0.421	-0.233	-0.283	-0.450	-0.116	-0.264	-0.577	0.099		0.030	-0.589	-0.168	-0.299	-0.047	-0.143	0.017	-0.072
LDL cholesterol	-0.118	-0.228	0.002	-0.107	-0.024	-0.197	-0.224	-0.002	-0.198	0.073	0.753	0.030		-0.018	0.743	-0.232	-0.015	-0.215	0.189	-0.022
VLDL	-0.229	-0.020	0.093	0.585	0.289	0.212	0.458	0.091	0.109	1.000	0.340	-0.589	-0.018		0.525	0.294	0.308	0.256	-0.128	0.153
Non-HDL-C	-0.087	-0.155	0.072	0.288	0.179	-0.057	0.102	0.029	-0.083	0.585	0.935	-0.168	0.743	0.525		-0.019	0.110	0.143	0.081	0.194
BoP(%)	0.123	-0.219	0.121	0.307	-0.003	-0.106	-0.006	-0.072	-0.025	0.250	-0.152	-0.299	-0.232	0.294	-0.019		-0.104	0.205	-0.294	-0.095
PD 4-5 mm	0.058	0.165	0.123	0.145	0.286	0.222	0.264	0.063	-0.149	0.254	0.089	-0.047	-0.015	0.308	0.110	-0.104		0.048	-0.034	0.161
PD ≥6 mm	0.113	-0.143	-0.329	0.256	0.195	0.420	0.440	0.246	0.133	0.247	0.118	-0.143	-0.215	0.256	0.143	0.205	0.048		-0.226	0.237
CAL 3-4 mm	-0.124	-0.007	-0.055	0.130	0.028	0.161	0.241	0.235	0.212	-0.080	0.157	0.017	0.189	-0.128	0.081	-0.294	-0.034	-0.226		0.216
CAL≥ 5mm	-0.059	0.032	-0.189	-0.001	0.064	0.130	0.195	-0.136	0.243	0.148	0.150	-0.072	-0.022	0.153	0.194	-0.095	0.161	0.237	0.216	

T2DM_without_P= type 2 Diabetes Mellitus without + Periodontitis; *SOD1*= Superoxide dismutase 1; *IRS2*= Insulin Receptor Substrate 2; HbA1c= Glycated hemoglobin; HOMA= Homeostasis model assessment; BMI= Body mass index; VLDL= Very low-density lipoprotein; BoP= Bleeding on probing; PD= Probing depth; CAL= Clinical attachment level. Values above light gray meant p values. The green colors meant that there was a positive significant correlation, and the red color meant negative significant correlation. Upper diagonal= p values. Lower diagonal values= p= Spearman’s correlation coefficients

Supplementary Figure S4- Correlation between SOD1 and IRS2 mRNA levels, SOD enzyme activity, demographic, physical, biochemical, and periodontal parameters (Group: Periodontitis-Baseline).

**Periodontitis Baseline**	*SOD1*	*IRS2*	*SOD (U/g)*	Fasting glucose	HbA1c (%)	Insulin (U/L)	HOMA	BMI	Waist-hip ratio	Triglycerides	Total cholesterol	HDL cholesterol	LDL cholesterol	VLDL	Non-HDL-C	BoP(%)	PD 4-5 mm	PD ≥6 mm	CAL 3-4 mm	CAL≥ 5mm
SOD1		0.584	-0.269	0.261	0.025	-0.137	-0.036	-0.183	-0.112	-0.108	0.158	0.196	0.106	-0.108	0.070	-0.410	-0.133	0.003	-0.252	-0.387
IRS2	0.584		-0.064	0.266	-0.047	-0.134	-0.069	-0.254	0.077	-0.103	0.134	0.109	0.159	-0.103	0.122	-0.535	-0.071	-0.120	0.052	-0.188
SOD (U/g)	-0.269	-0.064		-0.385	-0.020	-0.010	-0.093	0.079	0.110	-0.125	0.036	-0.098	0.141	-0.125	0.094	0.137	0.178	-0.121	0.517	0.420
Fasting glucose	0.261	0.266	-0.385		0.225	0.379	0.524	-0.119	0.176	0.330	0.258	-0.024	0.182	0.330	0.210	-0.357	-0.013	0.328	-0.297	-0.335
HbA1c (%)	0.025	-0.047	-0.020	0.225		0.134	0.145	0.135	0.262	0.212	0.327	-0.106	0.339	0.212	0.353	0.084	0.116	0.032	0.118	0.180
Insulin (U/L)	-0.137	-0.134	-0.010	0.379	0.134		0.970	0.470	0.040	0.403	0.134	-0.551	0.240	0.403	0.285	-0.135	0.111	0.355	0.038	0.023
HOMA	-0.036	-0.069	-0.093	0.524	0.145	0.970		0.402	0.060	0.401	0.194	-0.455	0.260	0.401	0.302	-0.212	0.071	0.372	-0.011	-0.084
BMI	-0.183	-0.254	0.079	-0.119	0.135	0.470	0.402		0.314	0.477	0.038	-0.506	0.075	0.477	0.204	0.341	0.297	0.343	0.070	0.369
Waist-hip ratio	-0.112	0.077	0.110	0.176	0.262	0.040	0.060	0.314		0.281	-0.156	-0.076	-0.243	0.281	-0.114	0.226	0.360	0.494	-0.097	0.364
Triglycerides	-0.108	-0.103	-0.125	0.330	0.212	0.403	0.401	0.477	0.281		0.462	-0.288	0.304	1.000	0.571	0.132	0.311	0.377	-0.236	0.152
Total cholesterol	0.158	0.134	0.036	0.258	0.327	0.134	0.194	0.038	-0.156	0.462		0.272	0.901	0.462	0.926	-0.025	0.042	0.070	-0.082	0.006
HDL cholesterol	0.196	0.109	-0.098	-0.024	-0.106	-0.551	-0.455	-0.506	-0.076	-0.288	0.272		0.004	-0.288	-0.062	-0.221	-0.195	-0.078	-0.150	-0.209
LDL cholesterol	0.106	0.159	0.141	0.182	0.339	0.240	0.260	0.075	-0.243	0.304	0.901	0.004		0.304	0.944	0.008	-0.007	-0.042	0.106	0.063
VLDL	-0.108	-0.103	-0.125	0.330	0.212	0.403	0.401	0.477	0.281	1.000	0.462	-0.288	0.304		0.571	0.132	0.311	0.377	-0.236	0.152
Non-HDL-C	0.070	0.122	0.094	0.210	0.353	0.285	0.302	0.204	-0.114	0.571	0.926	-0.062	0.944	0.571		0.064	0.102	0.062	0.019	0.124
BoP(%)	-0.410	-0.535	0.137	-0.357	0.084	-0.135	-0.212	0.341	0.226	0.132	-0.025	-0.221	0.008	0.132	0.064		0.442	0.240	-0.082	0.472
PD 4-5 mm	-0.133	-0.071	0.178	-0.013	0.116	0.111	0.071	0.297	0.360	0.311	0.042	-0.195	-0.007	0.311	0.102	0.442		0.562	0.155	0.509
PD ≥6 mm	0.003	-0.120	-0.121	0.328	0.032	0.355	0.372	0.343	0.494	0.377	0.070	-0.078	-0.042	0.377	0.062	0.240	0.562		-0.226	0.315
CAL 3-4 mm	-0.252	0.052	0.517	-0.297	0.118	0.038	-0.011	0.070	-0.097	-0.236	-0.082	-0.150	0.106	-0.236	0.019	-0.082	0.155	-0.226		0.510
CAL≥ 5mm	-0.387	-0.188	0.420	-0.335	0.180	0.023	-0.084	0.369	0.364	0.152	0.006	-0.209	0.063	0.152	0.124	0.472	0.509	0.315	0.510	

SOD1= Superoxide dismutase 1; *IRS2*= Insulin Receptor Substrate 2; HbA1c= Glycated hemoglobin; HOMA= Homeostasis model assessment; BMI= Body mass index; VLDL= Very low-density lipoprotein; BoP= Bleeding on probing; PD= Probing depth; CAL= Clinical attachment level. Values above light gray meant p values. The green colors meant that there was a positive significant correlation, and the red color meant negative significant correlation. Upper diagonal= p values. Lower diagonal values= p= Spearman's correlation coefficients

Supplementary Figure S5- Correlation between SOD1 and IRS2 mRNA levels, SOD enzyme activity, demographic, physical, biochemical, and periodontal parameters (Group: Control-Baseline).

**Control Baseline**	*SOD1*	*IRS2*	SOD (U/g)	Fasting glucose	HbA1c (%)	Insulin (U/L)	HOMA	BMI	Waist-hip ratio	Triglycerides	Total cholesterol	HDL cholesterol	LDL cholesterol	VLDL	Non-HDL-C	BoP(%)	PD 4-5 mm	PD ≥6 mm	CAL 3-4 mm	CAL≥ 5mm
SOD1		0.489	-0.211	0.176	-0.062	0.121	0.157	-0.078	-0.285	0.033	-0.097	0.064	-0.118	0.033	-0.080	-0.339	-0.105	-0.041	0.187	-0.367
IRS2	0.489		-0.135	0.057	0.135	-0.078	-0.068	-0.188	-0.208	-0.013	-0.047	0.143	-0.002	-0.013	0.083	-0.217	-0.085	0.163	0.158	0.025
SOD (U/g)	-0.211	-0.135		0.073	-0.306	-0.478	-0.476	-0.368	-0.053	-0.037	0.203	0.347	-0.120	-0.037	-0.066	-0.058	-0.019	0.118	-0.262	0.185
Fasting glucose	0.176	0.057	0.073		0.466	0.254	0.369	0.283	0.469	0.344	0.161	-0.188	0.062	0.344	0.182	-0.103	0.230	-0.020	-0.064	-0.145
HbA1c (%)	-0.062	0.135	-0.306	0.466		0.351	0.391	0.275	0.477	0.295	0.002	-0.267	0.105	0.295	0.195	0.115	0.308	-0.137	0.060	0.169
Insulin (U/L)	0.121	-0.078	-0.478	0.254	0.351		0.984	0.574	0.373	0.499	0.081	-0.444	0.074	0.499	0.187	0.003	0.165	-0.302	0.118	-0.047
HOMA	0.157	-0.068	-0.476	0.369	0.391	0.984		0.590	0.395	0.524	0.058	-0.455	0.055	0.524	0.174	-0.050	0.185	-0.302	0.084	-0.042
BMI	-0.078	-0.188	-0.368	0.283	0.275	0.574	0.590		0.324	0.404	0.003	-0.593	0.088	0.404	0.164	0.103	0.116	0.068	-0.029	0.031
Waist-hip ratio	-0.285	-0.208	-0.053	0.469	0.477	0.373	0.395	0.324		0.385	0.230	-0.244	0.205	0.385	0.270	0.295	0.407	0.011	0.074	0.211
Triglycerides	0.033	-0.013	-0.037	0.344	0.295	0.499	0.524	0.404	0.385		0.190	-0.376	-0.074	1.000	0.285	-0.029	0.019	-0.302	-0.026	0.105
Total cholesterol	-0.097	-0.047	0.203	0.161	0.002	0.081	0.058	0.003	0.230	0.190		-0.030	0.736	0.190	0.791	0.140	-0.150	-0.165	0.097	-0.034
HDL cholesterol	0.064	0.143	0.347	-0.188	-0.267	-0.444	-0.455	-0.593	-0.244	-0.376	-0.030		-0.444	-0.376	-0.512	0.071	-0.183	0.097	-0.134	-0.338
LDL cholesterol	-0.118	-0.002	-0.120	0.062	0.105	0.074	0.055	0.088	0.205	-0.074	0.736	-0.444		-0.074	0.913	0.050	-0.040	-0.029	0.194	0.241
VLDL	0.033	-0.013	-0.037	0.344	0.295	0.499	0.524	0.404	0.385	1.000	0.190	-0.376	-0.074		0.285	-0.029	0.019	-0.302	-0.026	0.105
Non-HDL-C	-0.080	0.083	-0.066	0.182	0.195	0.187	0.174	0.164	0.270	0.285	0.791	-0.512	0.913	0.285		-0.006	-0.059	-0.127	0.137	0.219
BoP(%)	-0.339	-0.217	-0.058	-0.103	0.115	0.003	-0.050	0.103	0.295	-0.029	0.140	0.071	0.050	-0.029	-0.006		0.008	-0.117	-0.046	-0.018
PD 4-5 mm	-0.105	-0.085	-0.019	0.230	0.308	0.165	0.185	0.116	0.407	0.019	-0.150	-0.183	-0.040	0.019	-0.059	0.008		0.071	0.283	0.073
PD ≥6 mm	-0.041	0.163	0.118	-0.020	-0.137	-0.302	-0.302	0.068	0.011	-0.302	-0.165	0.097	-0.029	-0.302	-0.127	-0.117	0.071		-0.205	-0.086
CAL 3-4 mm	0.187	0.158	-0.262	-0.064	0.060	0.118	0.084	-0.029	0.074	-0.026	0.097	-0.134	0.194	-0.026	0.137	-0.046	0.283	-0.205		0.187
CAL≥ 5mm	-0.367	0.025	0.185	-0.145	0.169	-0.047	-0.042	0.031	0.211	0.105	-0.034	-0.338	0.241	0.105	0.219	-0.018	0.073	-0.086	0.187	

SOD1= Superoxide dismutase 1; *IRS2*= Insulin Receptor Substrate 2; HbA1c= Glycated hemoglobin; HOMA= Homeostasis model assessment; BMI= Body mass index; VLDL= Very low-density lipoprotein; BoP= Bleeding on probing; PD= Probing depth; CAL= Clinical attachment level. Values above light gray meant p values. The green colors meant that there was a positive significant correlation, and the red color meant negative significant correlation. Upper diagonal= p values. Lower diagonal values= p= Spearman's correlation coefficients

Supplementary Figure S6- Correlation between SOD1 and IRS2 mRNA levels, SOD enzyme activity, demographic, physical, biochemical, and periodontal parameters (Group: T2DM_poorlyC+P- 90 days).

**T2DM_poorlyC+P 90 days**	*SOD1*	*IRS2*	*SOD (U/g)*	Fasting glucose	HbA1c (%)	Insulin (U/L)	HOMA	Triglycerides	Total cholesterol	HDL cholesterol	LDL cholesterol	VLDL	Non-HDL-C	BoP(%)	PD 4-5 mm	PD ≥6 mm	CAL 3-4 mm	CAL≥ 5mm
SOD1		0.385	-0.047	0.115	0.009	0.253	0.287	0.148	0.269	-0.044	0.337	0.148	0.324	0.019	0.184	0.079	0.465	0.120
IRS2	0.385		0.329	0.091	-0.178	-0.190	-0.174	0.020	0.050	0.057	0.069	0.020	0.038	-0.088	0.236	0.379	0.412	-0.035
SOD (U/g)	-0.047	0.329		-0.142	-0.186	0.179	0.026	-0.109	-0.230	0.060	-0.179	-0.109	-0.248	-0.003	0.005	0.151	-0.179	-0.072
Fasting glucose	0.115	0.091	-0.142		0.534	-0.241	0.231	0.206	0.192	-0.102	0.167	0.206	0.216	0.055	-0.025	0.349	0.087	0.203
HbA1c (%)	0.009	-0.178	-0.186	0.534		0.145	0.347	0.027	0.023	-0.163	0.096	0.027	0.080	0.077	-0.198	-0.065	-0.145	-0.040
Insulin (U/L)	0.253	-0.190	0.179	-0.241	0.145		0.853	0.074	-0.052	-0.152	-0.043	0.074	-0.014	0.141	0.110	-0.295	-0.018	0.113
HOMA	0.287	-0.174	0.026	0.231	0.347	0.853		0.280	0.038	-0.227	-0.025	0.280	0.082	0.110	0.123	-0.146	0.067	0.255
Triglycerides	0.148	0.020	-0.109	0.206	0.027	0.074	0.280		0.530	-0.038	0.199	1.000	0.544	0.056	-0.007	-0.074	0.284	-0.034
Total cholesterol	0.269	0.050	-0.230	0.192	0.023	-0.052	0.038	0.530		0.418	0.843	0.530	0.945	0.144	-0.345	-0.280	0.127	-0.494
HDL cholesterol	-0.044	0.057	0.060	-0.102	-0.163	-0.152	-0.227	-0.038	0.418		0.182	-0.038	0.180	-0.160	-0.340	-0.095	-0.225	-0.357
LDL cholesterol	0.337	0.069	-0.179	0.167	0.096	-0.043	-0.025	0.199	0.843	0.182		0.199	0.906	0.180	-0.352	-0.341	0.074	-0.553
VLDL	0.148	0.020	-0.109	0.206	0.027	0.074	0.280	1.000	0.530	-0.038	0.199		0.544	0.056	-0.007	-0.074	0.284	-0.034
Non-HDL-C	0.324	0.038	-0.248	0.216	0.080	-0.014	0.082	0.544	0.945	0.180	0.906	0.544		0.218	-0.260	-0.294	0.171	-0.478
BoP(%)	0.019	-0.088	-0.003	0.055	0.077	0.141	0.110	0.056	0.144	-0.160	0.180	0.056	0.218		0.375	-0.061	0.205	0.043
PD 4-5 mm	0.184	0.236	0.005	-0.025	-0.198	0.110	0.123	-0.007	-0.345	-0.340	-0.352	-0.007	-0.260	0.375		0.498	0.556	0.576
PD ≥6 mm	0.079	0.379	0.151	0.349	-0.065	-0.295	-0.146	-0.074	-0.280	-0.095	-0.341	-0.074	-0.294	-0.061	0.498		0.064	0.591
CAL 3-4 mm	0.465	0.412	-0.179	0.087	-0.145	-0.018	0.067	0.284	0.127	-0.225	0.074	0.284	0.171	0.205	0.556	0.064		-0.006
CAL≥ 5mm	0.120	-0.035	-0.072	0.203	-0.040	0.113	0.255	-0.034	-0.494	-0.357	-0.553	-0.034	-0.478	0.043	0.576	0.591	-0.006	

T2DM_poorlyC+P= type 2 Diabetes Mellitus poorly Controlled + Periodontitis; *SOD1*= Superoxide dismutase 1; *IRS2*= Insulin Receptor Substrate 2; HbA1c= Glycated hemoglobin; HOMA= Homeostasis model assessment; BMI= Body mass index; VLDL= Very low-density lipoprotein; BoP= Bleeding on probing; PD= Probing depth; CAL= Clinical attachment level. Values above light gray meant p values. The green colors meant that there was a positive significant correlation, and the red color meant negative significant correlation. Upper diagonal= p values. Lower diagonal values= p= Spearman's correlation coefficients

Supplementary Figure S7- Correlation between SOD1 and IRS2 mRNA levels, SOD enzyme activity, demographic, physical, biochemical, and periodontal parameters (Group: T2DM_wellC+P-90 days).

**T2DM_wellC+P 90 days**	*SOD1*	*IRS2*	SOD (U/g)	Fasting glucose	HbA1c (%)	Insulin (U/L)	HOMA	Triglycerides	Total cholesterol	HDL cholesterol	LDL cholesterol	VLDL	Non-HDL-C	BoP(%)	PD 4-5 mm	PD ≥6 mm	CAL 3-4 mm	CAL≥ 5mm
SOD1		0.346	-0.005	-0.236	-0.057	-0.321	-0.214	-0.296	0.182	-0.121	0.274	-0.296	0.128	0.157	-0.060	-0.290	-0.001	-0.339
IRS2	0.346		0.004	-0.169	-0.089	-0.494	-0.476	-0.362	-0.184	0.038	-0.053	-0.362	-0.220	0.033	-0.137	-0.111	-0.049	-0.136
SOD (U/g)	-0.005	0.004		-0.231	-0.201	0.118	0.094	0.168	0.094	-0.202	0.134	0.168	0.168	0.236	-0.030	-0.043	0.212	-0.089
Fasting glucose	-0.236	-0.169	-0.231		0.576	0.374	0.479	0.392	-0.019	0.077	-0.178	0.392	-0.037	0.184	0.260	0.259	0.241	0.174
HbA1c (%)	-0.057	-0.089	-0.201	0.576		0.300	0.291	0.095	-0.195	0.076	-0.293	0.095	-0.212	0.090	0.234	0.434	0.032	0.379
Insulin (U/L)	-0.321	-0.494	0.118	0.374	0.300		0.960	0.321	-0.193	-0.158	-0.299	0.321	-0.185	-0.048	-0.015	0.123	-0.041	-0.003
HOMA	-0.214	-0.476	0.094	0.479	0.291	0.960		0.412	-0.104	-0.082	-0.249	0.412	-0.098	-0.054	0.013	0.052	0.027	-0.074
Triglycerides	-0.296	-0.362	0.168	0.392	0.095	0.321	0.412		0.207	-0.112	-0.014	1.000	0.264	0.172	0.146	0.011	0.071	0.120
Total cholesterol	0.182	-0.184	0.094	-0.019	-0.195	-0.193	-0.104	0.207		0.243	0.843	0.207	0.891	-0.089	-0.019	-0.099	-0.017	0.011
HDL cholesterol	-0.121	0.038	-0.202	0.077	0.076	-0.158	-0.082	-0.112	0.243		-0.089	-0.112	-0.146	-0.045	-0.002	0.127	-0.044	0.042
LDL cholesterol	0.274	-0.053	0.134	-0.178	-0.293	-0.299	-0.249	-0.014	0.843	-0.089		-0.014	0.925	-0.091	0.056	-0.059	-0.026	0.043
VLDL	-0.296	-0.362	0.168	0.392	0.095	0.321	0.412	1.000	0.207	-0.112	-0.014		0.264	0.172	0.146	0.011	0.071	0.120
Non-HDL-C	0.128	-0.220	0.168	-0.037	-0.212	-0.185	-0.098	0.264	0.891	-0.146	0.925	0.264		-0.058	0.052	-0.058	-0.036	0.072
BoP(%)	0.157	0.033	0.236	0.184	0.090	-0.048	-0.054	0.172	-0.089	-0.045	-0.091	0.172	-0.058		0.324	0.225	0.246	0.176
PD 4-5 mm	-0.060	-0.137	-0.030	0.260	0.234	-0.015	0.013	0.146	-0.019	-0.002	0.056	0.146	0.052	0.324		0.632	0.473	0.509
PD ≥6 mm	-0.290	-0.111	-0.043	0.259	0.434	0.123	0.052	0.011	-0.099	0.127	-0.059	0.011	-0.058	0.225	0.632		0.140	0.715
CAL 3-4 mm	-0.001	-0.049	0.212	0.241	0.032	-0.041	0.027	0.071	-0.017	-0.044	-0.026	0.071	-0.036	0.246	0.473	0.140		0.220
CAL≥ 5mm	-0.339	-0.136	-0.089	0.174	0.379	-0.003	-0.074	0.120	0.011	0.042	0.043	0.120	0.072	0.176	0.509	0.715	0.220	

T2DM_wellC+P= type 2 Diabetes Mellitus well Controlled + Periodontitis; *SOD1*= Superoxide dismutase 1; *IRS2*= Insulin Receptor Substrate 2; HbA1c= Glycated hemoglobin; HOMA= Homeostasis model assessment; BMI= Body mass index; VLDL= Very low-density lipoprotein; BoP= Bleeding on probing; PD= Probing depth; CAL= Clinical attachment level. Values above light gray meant p values. The green colors meant that there was a positive significant correlation, and the red color meant negative significant correlation. Upper diagonal= p values. Lower diagonal values= p= Spearman's correlation coefficients.

Supplementary Figure S8- Correlation between SOD1 and IRS2 mRNA levels, SOD enzyme activity, demographic, physical, biochemical, and periodontal parameters (Group: T2DM_without_P-90 days).

**T2DM_without_P 90 days**	*SOD1*	*IRS2*	SOD (U/g)	Fasting glucose	HbA1c (%)	Insulin (U/L)	HOMA	Triglycerides	Total cholesterol	HDL cholesterol	LDL cholesterol	VLDL	Non-HDL-C	BoP(%)	PD 4-5 mm	PD ≥6 mm	CAL 3-4 mm	CAL≥ 5mm
SOD1		0.402	-0.082	0.089	0.045	-0.311	-0.288	-0.157	0.340	0.190	0.439	-0.157	0.405	-0.036	-0.204	-0.133	0.033	0.016
IRS2	0.402		0.253	0.103	0.060	-0.132	0.034	-0.191	0.367	0.566	0.243	-0.191	0.221	-0.059	0.040	0.051	-0.049	-0.086
SOD (U/g)	-0.082	0.253		0.001	0.010	0.088	0.113	0.029	-0.003	0.194	-0.196	0.029	-0.084	0.098	0.357	0.226	-0.195	-0.205
Fasting glucose	0.089	0.103	0.001		0.695	-0.068	0.416	0.454	0.095	-0.244	-0.028	0.454	0.232	0.063	0.135	-0.036	-0.135	-0.040
HbA1c (%)	0.045	0.060	0.010	0.695		-0.086	0.266	0.244	-0.178	-0.205	-0.295	0.244	-0.073	0.137	0.194	0.103	-0.263	0.008
Insulin (U/L)	-0.311	-0.132	0.088	-0.068	-0.086		0.819	0.296	-0.073	-0.359	-0.155	0.296	-0.047	0.561	0.113	0.266	0.062	0.045
HOMA	-0.288	0.034	0.113	0.416	0.266	0.819		0.548	0.083	-0.347	-0.090	0.548	0.152	0.608	0.270	0.225	0.040	0.151
Triglycerides	-0.157	-0.191	0.029	0.454	0.244	0.296	0.548		0.264	-0.458	-0.016	1.000	0.454	0.424	0.040	-0.276	-0.083	0.024
Total cholesterol	0.340	0.367	-0.003	0.095	-0.178	-0.073	0.083	0.264		0.387	0.848	0.264	0.900	0.096	0.005	-0.179	0.130	0.174
HDL cholesterol	0.190	0.566	0.194	-0.244	-0.205	-0.359	-0.347	-0.458	0.387		0.315	-0.458	0.039	-0.329	0.102	-0.051	0.053	0.073
LDL cholesterol	0.439	0.243	-0.196	-0.028	-0.295	-0.155	-0.090	-0.016	0.848	0.315		-0.016	0.837	-0.017	-0.030	-0.102	0.310	0.188
VLDL	-0.157	-0.191	0.029	0.454	0.244	0.296	0.548	1.000	0.264	-0.458	-0.016		0.454	0.424	0.040	-0.276	-0.083	0.024
Non-HDL-C	0.405	0.221	-0.084	0.232	-0.073	-0.047	0.152	0.454	0.900	0.039	0.837	0.454		0.201	-0.077	-0.205	0.134	0.107
BoP(%)	-0.036	-0.059	0.098	0.063	0.137	0.561	0.608	0.424	0.096	-0.329	-0.017	0.424	0.201		0.271	0.143	-0.035	0.122
PD 4-5 mm	-0.204	0.040	0.357	0.135	0.194	0.113	0.270	0.040	0.005	0.102	-0.030	0.040	-0.077	0.271		0.302	0.205	0.201
PD ≥6 mm	-0.133	0.051	0.226	-0.036	0.103	0.266	0.225	-0.276	-0.179	-0.051	-0.102	-0.276	-0.205	0.143	0.302		-0.221	0.148
CAL 3-4 mm	0.033	-0.049	-0.195	-0.135	-0.263	0.062	0.040	-0.083	0.130	0.053	0.310	-0.083	0.134	-0.035	0.205	-0.221		0.581
CAL≥ 5mm	0.016	-0.086	-0.205	-0.040	0.008	0.045	0.151	0.024	0.174	0.073	0.188	0.024	0.107	0.122	0.201	0.148	0.581	

T2DM_without_P= type 2 Diabetes Mellitus without+ Periodontitis; *SOD1*= Superoxide dismutase 1; *IRS2*= Insulin Receptor Substrate 2; HbA1c= Glycated hemoglobin; HOMA= Homeostasis model assessment; BMI= Body mass index; VLDL= Very low-density lipoprotein; BoP= Bleeding on probing; PD= Probing depth; CAL= Clinical attachment level. Values above light gray meant p values. The green colors meant that there was a positive significant correlation, and the red color meant negative significant correlation. Upper diagonal= p values. Lower diagonal values= p= Spearman's correlation coefficients.

Supplementary Figure S9- Correlation between SOD1 and IRS2 mRNA levels, SOD enzyme activity, demographic, physical, biochemical, and periodontal parameters (Group: Periodontitis- 90 days).

**Periodontitis 90 days**	*SOD1*	*IRS2*	SOD (U/g)	Fasting glucose	HbA1c (%)	Insulin (U/L)	HOMA	Triglycerides	Total cholesterol	HDL cholesterol	LDL cholesterol	VLDL	Non-HDL-C	BoP(%)	PD 4-5 mm	PD ≥6 mm	CAL 3-4 mm	CAL≥ 5mm
SOD1		0.365	0.348	-0.253	0.064	0.093	0.083	0.247	0.266	0.011	0.183	0.235	0.301	-0.063	-0.099	-0.054	-0.089	-0.272
IRS2	0.365		0.001	-0.063	-0.378	-0.038	-0.056	0.176	0.197	0.056	0.080	0.166	0.160	-0.174	0.013	0.029	-0.121	-0.026
SOD (U/g)	0.348	0.001		-0.101	0.267	0.157	0.176	-0.193	-0.074	0.098	0.040	-0.193	-0.087	0.048	0.007	0.108	0.024	-0.062
Fasting glucose	-0.253	-0.063	-0.101		0.112	0.323	0.428	0.430	0.158	-0.212	0.190	0.430	0.311	0.054	-0.044	0.004	-0.429	-0.022
HbA1c (%)	0.064	-0.378	0.267	0.112		0.368	0.378	0.117	0.269	-0.099	0.269	0.117	0.278	0.235	-0.006	-0.276	0.006	-0.188
Insulin (U/L)	0.093	-0.038	0.157	0.323	0.368		0.987	0.475	0.047	-0.450	0.095	0.475	0.246	-0.023	0.190	0.205	-0.234	0.048
HOMA	0.083	-0.056	0.176	0.428	0.378	0.987		0.518	0.067	-0.467	0.198	0.563	0.350	0.037	0.256	0.246	-0.172	0.093
Triglycerides	0.247	0.176	-0.193	0.430	0.117	0.475	0.518		0.304	-0.371	0.054	1.000	0.455	0.097	0.166	0.186	-0.237	0.105
Total cholesterol	0.266	0.197	-0.074	0.158	0.269	0.047	0.067	0.304		0.189	0.803	0.304	0.884	0.060	-0.045	-0.075	0.045	-0.237
HDL cholesterol	0.011	0.056	0.098	-0.212	-0.099	-0.450	-0.467	-0.371	0.189		-0.114	-0.371	-0.185	-0.318	-0.258	-0.002	-0.091	-0.069
LDL cholesterol	0.183	0.080	0.040	0.190	0.269	0.095	0.198	0.054	0.803	-0.114		0.143	0.884	0.230	-0.009	-0.132	0.095	-0.306
VLDL	0.235	0.166	-0.193	0.430	0.117	0.475	0.563	1.000	0.304	-0.371	0.143		0.506	0.126	0.244	0.234	-0.135	0.157
Non-HDL-C	0.301	0.160	-0.087	0.311	0.278	0.246	0.350	0.455	0.884	-0.185	0.884	0.506		0.274	0.129	0.034	0.054	-0.157
BoP(%)	-0.063	-0.174	0.048	0.054	0.235	-0.023	0.037	0.097	0.060	-0.318	0.230	0.126	0.274		0.573	0.410	0.170	0.471
PD 4-5 mm	-0.099	0.013	0.007	-0.044	-0.006	0.190	0.256	0.166	-0.045	-0.258	-0.009	0.244	0.129	0.573		0.696	0.357	0.676
PD ≥6 mm	-0.054	0.029	0.108	0.004	-0.276	0.205	0.246	0.186	-0.075	-0.002	-0.132	0.234	0.034	0.410	0.696		0.171	0.793
CAL 3-4 mm	-0.089	-0.121	0.024	-0.429	0.006	-0.234	-0.172	-0.237	0.045	-0.091	0.095	-0.135	0.054	0.170	0.357	0.171		0.374
CAL≥ 5mm	-0.272	-0.026	-0.062	-0.022	-0.188	0.048	0.093	0.105	-0.237	-0.069	-0.306	0.157	-0.157	0.471	0.676	0.793	0.374	

SOD1= Superoxide dismutase 1; *IRS2*= Insulin Receptor Substrate 2; HbA1c= Glycated hemoglobin; HOMA= Homeostasis model assessment; BMI= Body mass index; VLDL= Very low-density lipoprotein; BoP= Bleeding on probing; PD= Probing depth; CAL= Clinical attachment level. Values above light gray meant p values. The green colors meant that there was a positive significant correlation, and the red color meant negative significant correlation. Upper diagonal= p values. Lower diagonal values= p= Spearman's correlation coefficients.

Supplementary Figure S10- Correlation between SOD1 and IRS2 mRNA levels, SOD enzyme activity, demographic, physical, biochemical, and periodontal parameters (Group:Control-90 days).

**Control 90 days**	*SOD1*	*IRS2*	SOD (U/g)	Fasting glucose	HbA1c (%)	Insulin (U/L)	HOMA	Triglycerides	Total cholesterol	HDL cholesterol	LDL cholesterol	VLDL	Non-HDL-C	BoP(%)	PD 4-5 mm	PD ≥6 mm	CAL 3-4 mm	CAL≥ 5mm
SOD1		0.415	0.410	-0.323	0.094	-0.177	-0.218	-0.113	-0.118	-0.089	-0.050	-0.113	-0.074	-0.382	-0.078	-0.015	0.055	-0.062
IRS2	0.415		0.413	0.150	-0.257	-0.136	-0.040	-0.468	-0.072	0.148	0.048	-0.468	-0.173	-0.010	-0.319	-0.184	-0.010	-0.127
SOD (U/g)	0.410	0.413		-0.068	0.340	-0.053	-0.089	-0.237	-0.483	-0.082	-0.342	-0.237	-0.400	-0.169	-0.131	-0.056	-0.202	-0.105
Fasting glucose	-0.323	0.150	-0.068		0.105	-0.064	0.282	-0.066	-0.015	-0.045	0.136	-0.066	0.040	0.273	0.120	0.322	0.193	0.190
HbA1c (%)	0.094	-0.257	0.340	0.105		0.135	0.155	0.249	-0.104	-0.061	-0.193	0.249	-0.094	-0.048	0.036	0.014	0.056	0.147
Insulin (U/L)	-0.177	-0.136	-0.053	-0.064	0.135		0.909	0.212	0.100	-0.207	0.160	0.212	0.259	0.289	-0.026	-0.224	0.186	-0.084
HOMA	-0.218	-0.040	-0.089	0.282	0.155	0.909		0.161	0.196	-0.203	0.307	0.161	0.344	0.344	0.011	-0.133	0.285	0.003
Triglycerides	-0.113	-0.468	-0.237	-0.066	0.249	0.212	0.161		0.317	-0.405	0.159	1.000	0.500	0.141	-0.022	0.042	0.049	0.074
Total cholesterol	-0.118	-0.072	-0.483	-0.015	-0.104	0.100	0.196	0.317		0.076	0.785	0.317	0.777	0.155	-0.253	0.056	0.065	-0.005
HDL cholesterol	-0.089	0.148	-0.082	-0.045	-0.061	-0.207	-0.203	-0.405	0.076		-0.394	-0.405	-0.527	0.032	-0.051	-0.126	0.067	0.002
LDL cholesterol	-0.050	0.048	-0.342	0.136	-0.193	0.160	0.307	0.159	0.785	-0.394		0.159	0.915	0.155	-0.116	0.070	0.042	-0.015
VLDL	-0.113	-0.468	-0.237	-0.066	0.249	0.212	0.161	1.000	0.317	-0.405	0.159		0.500	0.141	-0.022	0.042	0.049	0.074
Non-HDL-C	-0.074	-0.173	-0.400	0.040	-0.094	0.259	0.344	0.500	0.777	-0.527	0.915	0.500		0.124	-0.071	0.056	0.024	-0.062
BoP(%)	-0.382	-0.010	-0.169	0.273	-0.048	0.289	0.344	0.141	0.155	0.032	0.155	0.141	0.124		0.193	0.028	0.029	0.158
PD 4-5 mm	-0.078	-0.319	-0.131	0.120	0.036	-0.026	0.011	-0.022	-0.253	-0.051	-0.116	-0.022	-0.071	0.193		0.065	0.302	0.328
PD ≥6 mm	-0.015	-0.184	-0.056	0.322	0.014	-0.224	-0.133	0.042	0.056	-0.126	0.070	0.042	0.056	0.028	0.065		0.098	0.138
CAL 3-4 mm	0.055	-0.010	-0.202	0.193	0.056	0.186	0.285	0.049	0.065	0.067	0.042	0.049	0.024	0.029	0.302	0.098		0.548
CAL≥ 5mm	-0.062	-0.127	-0.105	0.190	0.147	-0.084	0.003	0.074	-0.005	0.002	-0.015	0.074	-0.062	0.158	0.328	0.138	0.548	

SOD1= Superoxide dismutase 1; *IRS2*= Insulin Receptor Substrate 2; HbA1c= Glycated hemoglobin; HOMA= Homeostasis model assessment; BMI= Body mass index; VLDL= Very low-density lipoprotein; BoP= Bleeding on probing; PD= Probing depth; CAL= Clinical attachment level. Values above light gray meant p values. The green colors meant that there was a positive significant correlation, and the red color meant negative significant correlation. Upper diagonal= p values. Lower diagonal values= p= Spearman's correlation coefficients.

Supplementary Figure S11- Correlation between SOD1 and IRS2 mRNA levels, SOD enzyme activity, demographic, physical, biochemical, and periodontal parameters (Group:T2DM_poorlyC+P-180 days).

**T2DM_poorlyC+P 180 days**	*SOD1*	*IRS2*	*SOD (U/g)*	Fasting glucose	HbA1c (%)	Insulin (U/L)	HOMA	Triglycerides	Total cholesterol	HDL cholesterol	LDL cholesterol	VLDL	Non-HDL-C	BoP(%)	PD 4-5 mm	PD ≥6 mm	CAL 3-4 mm	CAL≥ 5mm
SOD1		0.119	-0.309	0.215	0.290	0.047	0.208	0.266	0.257	0.090	0.109	0.266	0.193	0.477	0.257	-0.002	0.253	-0.264
IRS2	0.119		0.086	-0.154	0.029	-0.208	0.024	0.336	-0.191	-0.117	-0.220	0.336	-0.147	0.187	0.042	-0.181	0.199	-0.265
SOD (U/g)	-0.309	0.086		0.049	-0.015	-0.127	-0.028	0.044	0.110	0.127	-0.021	0.044	0.050	-0.133	0.031	-0.097	0.024	0.423
Fasting glucose	0.215	-0.154	0.049		0.653	0.131	0.406	0.204	0.392	0.197	0.254	0.204	0.347	0.190	0.057	-0.034	0.017	-0.031
HbA1c (%)	0.290	0.029	-0.015	0.653		0.320	0.424	0.264	0.208	-0.046	0.105	0.264	0.168	0.153	0.004	-0.123	0.182	-0.417
Insulin (U/L)	0.047	-0.208	-0.127	0.131	0.320		0.509	0.068	0.279	-0.029	0.300	0.068	0.281	-0.160	-0.116	0.066	-0.219	-0.584
HOMA	0.208	0.024	-0.028	0.406	0.424	0.509		0.428	0.324	0.012	0.159	0.428	0.263	0.266	-0.155	-0.280	-0.005	-0.114
Triglycerides	0.266	0.336	0.044	0.204	0.264	0.068	0.428		0.503	-0.024	0.306	1.000	0.544	0.470	-0.013	-0.084	0.238	-0.135
Total cholesterol	0.257	-0.191	0.110	0.392	0.208	0.279	0.324	0.503		0.350	0.902	0.503	0.943	0.169	-0.212	-0.068	-0.183	-0.135
HDL cholesterol	0.090	-0.117	0.127	0.197	-0.046	-0.029	0.012	-0.024	0.350		0.145	-0.024	0.101	-0.177	0.077	0.431	-0.104	-0.281
LDL cholesterol	0.109	-0.220	-0.021	0.254	0.105	0.300	0.159	0.306	0.902	0.145		0.306	0.942	0.042	-0.285	-0.126	-0.333	-0.136
VLDL	0.266	0.336	0.044	0.204	0.264	0.068	0.428	1.000	0.503	-0.024	0.306		0.544	0.470	-0.013	-0.084	0.238	-0.135
Non-HDL-C	0.193	-0.147	0.050	0.347	0.168	0.281	0.263	0.544	0.943	0.101	0.942	0.544		0.186	-0.305	-0.215	-0.241	-0.079
BoP(%)	0.477	0.187	-0.133	0.190	0.153	-0.160	0.266	0.470	0.169	-0.177	0.042	0.470	0.186		0.398	0.028	0.386	-0.155
PD 4-5 mm	0.257	0.042	0.031	0.057	0.004	-0.116	-0.155	-0.013	-0.212	0.077	-0.285	-0.013	-0.305	0.398		0.520	0.658	0.031
PD ≥6 mm	-0.002	-0.181	-0.097	-0.034	-0.123	0.066	-0.280	-0.084	-0.068	0.431	-0.126	-0.084	-0.215	0.028	0.520		0.102	-0.090
CAL 3-4 mm	0.253	0.199	0.024	0.017	0.182	-0.219	-0.005	0.238	-0.183	-0.104	-0.333	0.238	-0.241	0.386	0.658	0.102		0.080
CAL≥ 5mm	-0.264	-0.265	0.423	-0.031	-0.417	-0.584	-0.114	-0.135	-0.135	-0.281	-0.136	-0.135	-0.079	-0.155	0.031	-0.090	0.080	

T2DM_poorlyC+P= type 2 Diabetes Mellitus poorly Controlled + Periodontitis; *SOD1*= Superoxide dismutase 1; *IRS2*= Insulin Receptor Substrate 2; HbA1c= Glycated hemoglobin; HOMA= Homeostasis model assessment; BMI= Body mass index; VLDL= Very low-density lipoprotein; BoP= Bleeding on probing; PD= Probing depth; CAL= Clinical attachment level. Values above light gray meant p values. The green colors meant that there was a positive significant correlation, and the red color meant negative significant correlation. Upper diagonal= p values. Lower diagonal values= p= Spearman's correlation coefficients

Supplementary Figure S12- Correlation between SOD1 and IRS2 mRNA levels, SOD enzyme activity, demographic, physical, biochemical, and periodontal parameters (Group: T2DM_wellC+P- 180days).

**T2DM_wellC+P 180 days**	*SOD1*	*IRS2*	SOD (U/g)	Fasting glucose	HbA1c (%)	Insulin (U/L)	HOMA	Triglycerides	Total cholesterol	HDL cholesterol	LDL cholesterol	VLDL	Non-HDL-C	BoP(%)	PD 4-5 mm	PD ≥6 mm	CAL 3-4 mm	CAL≥ 5mm
SOD1		0.370	-0.042	0.072	0.068	-0.331	-0.175	-0.297	-0.297	-0.063	-0.177	-0.297	-0.302	0.286	0.285	0.109	0.286	0.010
IRS2	0.370		-0.062	-0.156	-0.037	0.034	-0.054	0.273	0.273	0.104	-0.156	0.273	-0.035	-0.013	-0.022	-0.243	-0.126	-0.026
SOD (U/g)	-0.042	-0.062		0.016	-0.290	0.103	0.054	0.065	0.065	-0.191	-0.106	0.065	0.128	0.127	-0.164	-0.024	0.374	-0.217
Fasting glucose	0.072	-0.156	0.016		0.692	0.280	0.659	0.146	0.146	-0.240	-0.051	0.146	0.042	0.393	0.452	0.437	0.372	0.310
HbA1c (%)	0.068	-0.037	-0.290	0.692		0.075	0.353	0.060	0.060	0.063	0.072	0.060	0.079	0.294	0.406	0.476	0.108	0.469
Insulin (U/L)	-0.331	0.034	0.103	0.280	0.075		0.880	0.351	0.351	-0.372	-0.168	0.351	0.034	-0.014	-0.122	-0.116	-0.011	-0.069
HOMA	-0.175	-0.054	0.054	0.659	0.353	0.880		0.280	0.280	-0.410	-0.214	0.280	-0.042	0.175	0.123	0.123	0.136	0.100
Triglycerides	-0.297	0.273	0.065	0.146	0.060	0.351	0.280		1.000	-0.287	0.194	1.000	0.672	-0.119	0.105	0.047	0.213	0.188
Total cholesterol	-0.297	0.273	0.065	0.146	0.060	0.351	0.280	1.000		-0.287	0.194	1.000	0.672	-0.119	0.105	0.047	0.213	0.188
HDL cholesterol	-0.063	0.104	-0.191	-0.240	0.063	-0.372	-0.410	-0.287	-0.287		0.209	-0.287	-0.036	-0.052	0.084	0.043	-0.211	0.068
LDL cholesterol	-0.177	-0.156	-0.106	-0.051	0.072	-0.168	-0.214	0.194	0.194	0.209		0.194	0.787	0.088	0.030	0.078	-0.101	0.050
VLDL	-0.297	0.273	0.065	0.146	0.060	0.351	0.280	1.000	1.000	-0.287	0.194		0.672	-0.119	0.105	0.047	0.213	0.188
Non-HDL-C	-0.302	-0.035	0.128	0.042	0.079	0.034	-0.042	0.672	0.672	-0.036	0.787	0.672		-0.075	-0.006	0.095	0.100	0.094
BoP(%)	0.286	-0.013	0.127	0.393	0.294	-0.014	0.175	-0.119	-0.119	-0.052	0.088	-0.119	-0.075		0.528	0.447	0.300	0.316
PD 4-5 mm	0.285	-0.022	-0.164	0.452	0.406	-0.122	0.123	0.105	0.105	0.084	0.030	0.105	-0.006	0.528		0.649	0.515	0.602
PD ≥6 mm	0.109	-0.243	-0.024	0.437	0.476	-0.116	0.123	0.047	0.047	0.043	0.078	0.047	0.095	0.447	0.649		0.194	0.709
CAL 3-4 mm	0.286	-0.126	0.374	0.372	0.108	-0.011	0.136	0.213	0.213	-0.211	-0.101	0.213	0.100	0.300	0.515	0.194		0.282
CAL≥ 5mm	0.010	-0.026	-0.217	0.310	0.469	-0.069	0.100	0.188	0.188	0.068	0.050	0.188	0.094	0.316	0.602	0.709	0.282	

T2DM_wellC+P= type 2 Diabetes Mellitus well Controlled + Periodontitis; *SOD1*= Superoxide dismutase 1; *IRS2*= Insulin Receptor Substrate 2; HbA1c= Glycated hemoglobin; HOMA= Homeostasis model assessment; BMI= Body mass index; VLDL= Very low-density lipoprotein; BoP= Bleeding on probing; PD= Probing depth; CAL= Clinical attachment level. Values above light gray meant p values. The green colors meant that there was a positive significant correlation, and the red color meant negative significant correlation. Upper diagonal= p values. Lower diagonal values= p= Spearman's correlation coefficients.

Supplementary Figure S13- Correlation between SOD1 and IRS2 mRNA levels, SOD enzyme activity, demographic, physical, biochemical, and periodontal parameters (Group: T2DM_without_P- 180 days).

**T2DM_without_P 180 days**	*SOD1*	*IRS2*	SOD (U/g)	Fasting glucose	HbA1c (%)	Insulin (U/L)	HOMA	Triglycerides	Total cholesterol	HDL cholesterol	LDL cholesterol	VLDL	Non-HDL-C	BoP(%)	PD 4-5 mm	PD ≥6 mm	CAL 3-4 mm	CAL≥ 5mm
SOD1		0.035	-0.042	0.169	0.226	-0.143	-0.003	0.106	0.106	-0.356	-0.133	0.106	-0.048	-0.192	0.090	0.090	0.097	-0.076
IRS2	0.035		0.283	0.236	0.143	0.099	0.143	0.261	0.261	-0.148	-0.177	0.261	-0.063	-0.102	0.050	0.050	0.175	0.030
SOD (U/g)	-0.042	0.283		0.251	0.060	-0.241	-0.154	-0.001	-0.001	-0.012	-0.019	-0.001	-0.009	-0.056	0.159	-0.032	0.264	-0.026
Fasting glucose	0.169	0.236	0.251		0.845	-0.148	0.391	0.453	0.453	-0.149	0.006	0.453	0.245	0.031	0.176	0.263	0.067	0.246
HbA1c (%)	0.226	0.143	0.060	0.845		0.018	0.469	0.411	0.411	-0.147	-0.044	0.411	0.161	0.188	0.115	0.166	-0.027	0.064
Insulin (U/L)	-0.143	0.099	-0.241	-0.148	0.018		0.828	0.286	0.286	-0.292	-0.047	0.286	0.019	0.233	0.025	-0.233	-0.031	-0.105
HOMA	-0.003	0.143	-0.154	0.391	0.469	0.828		0.531	0.531	-0.412	-0.083	0.531	0.155	0.319	0.130	-0.107	-0.035	0.025
Triglycerides	0.106	0.261	-0.001	0.453	0.411	0.286	0.531		1.000	-0.554	-0.086	1.000	0.404	0.309	0.502	0.185	0.272	0.267
Total cholesterol	0.106	0.261	-0.001	0.453	0.411	0.286	0.531	1.000		-0.554	-0.086	1.000	0.404	0.309	0.502	0.185	0.272	0.267
HDL cholesterol	-0.356	-0.148	-0.012	-0.149	-0.147	-0.292	-0.412	-0.554	-0.554		0.290	-0.554	0.041	-0.230	-0.412	-0.165	0.090	0.114
LDL cholesterol	-0.133	-0.177	-0.019	0.006	-0.044	-0.047	-0.083	-0.086	-0.086	0.290		-0.086	0.826	0.105	-0.266	-0.165	-0.057	0.367
VLDL	0.106	0.261	-0.001	0.453	0.411	0.286	0.531	1.000	1.000	-0.554	-0.086		0.404	0.309	0.502	0.185	0.272	0.267
Non-HDL-C	-0.048	-0.063	-0.009	0.245	0.161	0.019	0.155	0.404	0.404	0.041	0.826	0.404		0.325	-0.009	-0.126	0.067	0.420
BoP(%)	-0.192	-0.102	-0.056	0.031	0.188	0.233	0.319	0.309	0.309	-0.230	0.105	0.309	0.325		0.191	-0.146	-0.123	0.061
PD 4-5 mm	0.090	0.050	0.159	0.176	0.115	0.025	0.130	0.502	0.502	-0.412	-0.266	0.502	-0.009	0.191		0.330	0.240	0.125
PD ≥6 mm	0.090	0.050	-0.032	0.263	0.166	-0.233	-0.107	0.185	0.185	-0.165	-0.165	0.185	-0.126	-0.146	0.330		0.127	0.212
CAL 3-4 mm	0.097	0.175	0.264	0.067	-0.027	-0.031	-0.035	0.272	0.272	0.090	-0.057	0.272	0.067	-0.123	0.240	0.127		0.476
CAL≥ 5mm	-0.076	0.030	-0.026	0.246	0.064	-0.105	0.025	0.267	0.267	0.114	0.367	0.267	0.420	0.061	0.125	0.212	0.476	

T2DM_without_P= type 2 Diabetes Mellitus without Periodontitis; *SOD1*= Superoxide dismutase 1; *IRS2*= Insulin Receptor Substrate 2; HbA1c= Glycated hemoglobin; HOMA= Homeostasis model assessment; BMI= Body mass index; VLDL= Very low-density lipoprotein; BoP= Bleeding on probing; PD= Probing depth; CAL= Clinical attachment level. Values above light gray meant p values. The green colors meant that there was a positive significant correlation, and the red color meant negative significant correlation. Upper diagonal= p values. Lower diagonal values= p= Spearman's correlation coefficients.

Supplementary Figure S14- Correlation between SOD1 and IRS2 mRNA levels, SOD enzyme activity, demographic, physical, biochemical, and periodontal parameters (Group: Periodontitis- 180 days).

**Periodontitis 180 days**	*SOD1*	*IRS2*	SOD (U/g)	Fasting glucose	HbA1c (%)	Insulin (U/L)	HOMA	Triglycerides	Total cholesterol	HDL cholesterol	LDL cholesterol	VLDL	Non-HDL-C	BoP(%)	PD 4-5 mm	PD ≥6 mm	CAL 3-4 mm	CAL≥ 5mm
SOD1		0.633	-0.073	0.245	-0.003	0.029	0.065	0.140	0.140	-0.041	0.075	0.140	0.162	0.169	0.137	0.193	0.022	-0.029
IRS2	0.633		-0.416	0.099	0.255	0.108	0.114	0.118	0.118	0.047	0.070	0.118	0.145	0.132	-0.101	-0.056	0.052	0.008
SOD (U/g)	-0.073	-0.416		-0.086	-0.332	-0.373	-0.375	-0.500	-0.500	0.126	0.180	-0.500	-0.001	-0.483	-0.195	-0.077	-0.004	-0.301
Fasting glucose	0.245	0.099	-0.086		0.208	0.106	0.220	0.088	0.088	0.101	0.212	0.088	0.251	0.129	0.279	0.221	0.270	0.151
HbA1c (%)	-0.003	0.255	-0.332	0.208		-0.048	-0.038	-0.027	-0.027	0.077	0.157	-0.027	0.196	0.058	0.187	-0.108	0.361	0.045
Insulin (U/L)	0.029	0.108	-0.373	0.106	-0.048		0.989	0.500	0.500	-0.375	-0.091	0.500	0.109	0.095	0.159	0.222	-0.126	0.325
HOMA	0.065	0.114	-0.375	0.220	-0.038	0.989		0.513	0.513	-0.366	-0.059	0.513	0.140	0.122	0.200	0.250	-0.087	0.341
Triglycerides	0.140	0.118	-0.500	0.088	-0.027	0.500	0.513		1.000	-0.242	-0.287	1.000	0.040	0.216	0.332	0.294	-0.150	0.357
Total cholesterol	0.140	0.118	-0.500	0.088	-0.027	0.500	0.513	1.000		-0.242	-0.287	1.000	0.040	0.216	0.332	0.294	-0.150	0.357
HDL cholesterol	-0.041	0.047	0.126	0.101	0.077	-0.375	-0.366	-0.242	-0.242		-0.177	-0.242	-0.268	-0.081	-0.113	0.028	0.044	-0.052
LDL cholesterol	0.075	0.070	0.180	0.212	0.157	-0.091	-0.059	-0.287	-0.287	-0.177		-0.287	0.926	0.018	0.108	-0.060	-0.031	-0.261
VLDL	0.140	0.118	-0.500	0.088	-0.027	0.500	0.513	1.000	1.000	-0.242	-0.287		0.040	0.216	0.332	0.294	-0.150	0.357
Non-HDL-C	0.162	0.145	-0.001	0.251	0.196	0.109	0.140	0.040	0.040	-0.268	0.926	0.040		0.091	0.240	0.038	-0.077	-0.156
BoP(%)	0.169	0.132	-0.483	0.129	0.058	0.095	0.122	0.216	0.216	-0.081	0.018	0.216	0.091		0.626	0.414	0.118	0.494
PD 4-5 mm	0.137	-0.101	-0.195	0.279	0.187	0.159	0.200	0.332	0.332	-0.113	0.108	0.332	0.240	0.626		0.788	0.475	0.774
PD ≥6 mm	0.193	-0.056	-0.077	0.221	-0.108	0.222	0.250	0.294	0.294	0.028	-0.060	0.294	0.038	0.414	0.788		0.284	0.738
CAL 3-4 mm	0.022	0.052	-0.004	0.270	0.361	-0.126	-0.087	-0.150	-0.150	0.044	-0.031	-0.150	-0.077	0.118	0.475	0.284		0.492
CAL≥ 5mm	-0.029	0.008	-0.301	0.151	0.045	0.325	0.341	0.357	0.357	-0.052	-0.261	0.357	-0.156	0.494	0.774	0.738	0.492	

SOD1= Superoxide dismutase 1; *IRS2*= Insulin Receptor Substrate 2; HbA1c= Glycated hemoglobin; HOMA= Homeostasis model assessment; BMI= Body mass index; VLDL= Very low-density lipoprotein; BoP= Bleeding on probing; PD= Probing depth; CAL= Clinical attachment level. Values above light gray meant p values. The green colors meant that there was a positive significant correlation, and the red color meant negative significant correlation. Upper diagonal= p values. Lower diagonal values= p= Spearman's correlation coefficients.

Supplementary Figure S15- Correlation between SOD1 and IRS2 mRNA levels, SOD enzyme activity, demographic, physical, biochemical, and periodontal parameters (Group: Control- 180 days).

**Control 180 days**	*SOD1*	*IRS2*	SOD (U/g)	Fasting glucose	HbA1c (%)	Insulin (U/L)	HOMA	Triglycerides	Total cholesterol	HDL cholesterol	LDL cholesterol	VLDL	Non-HDL-C	BoP(%)	PD 4-5 mm	PD ≥6 mm	CAL 3-4 mm	CAL≥ 5mm
SOD1		0.408	-0.038	-0.159	0.008	-0.255	-0.239	-0.292	-0.252	0.266	-0.342	-0.305	-0.362	0.145	-0.014	-0.247	0.198	0.129
IRS2	0.408		-0.196	-0.061	-0.127	-0.221	-0.200	-0.064	0.071	0.289	-0.071	-0.028	-0.080	0.193	-0.216	-0.139	-0.013	0.100
SOD (U/g)	-0.038	-0.196		0.007	-0.078	-0.346	-0.282	-0.223	-0.151	0.210	-0.248	-0.192	-0.297	-0.069	-0.043	0.350	-0.129	-0.152
Fasting glucose	-0.159	-0.061	0.007		0.023	-0.051	-0.053	-0.201	-0.207	-0.011	-0.033	-0.199	-0.138	-0.036	0.255	0.392	0.171	0.228
HbA1c (%)	0.008	-0.127	-0.078	0.023		0.083	0.138	0.059	-0.063	0.184	-0.216	0.032	-0.148	-0.077	-0.127	-0.056	0.067	-0.256
Insulin (U/L)	-0.255	-0.221	-0.346	-0.051	0.083		0.972	0.548	0.264	-0.634	0.410	0.491	0.583	-0.102	-0.200	0.028	0.020	0.173
HOMA	-0.239	-0.200	-0.282	-0.053	0.138	0.972		0.518	0.275	-0.555	0.386	0.453	0.553	-0.158	-0.141	0.056	0.084	0.176
Triglycerides	-0.292	-0.064	-0.223	-0.201	0.059	0.548	0.518		0.185	-0.407	0.030	1.000	0.359	0.016	0.035	0.184	0.044	0.254
Total cholesterol	-0.252	0.071	-0.151	-0.207	-0.063	0.264	0.275	0.185		-0.024	0.825	0.195	0.829	-0.271	-0.164	0.147	-0.040	0.044
HDL cholesterol	0.266	0.289	0.210	-0.011	0.184	-0.634	-0.555	-0.407	-0.024		-0.402	-0.394	-0.535	0.140	0.162	-0.217	0.004	-0.157
LDL cholesterol	-0.342	-0.071	-0.248	-0.033	-0.216	0.410	0.386	0.030	0.825	-0.402		0.040	0.914	-0.387	-0.193	0.196	-0.041	0.099
VLDL	-0.305	-0.028	-0.192	-0.199	0.032	0.491	0.453	1.000	0.195	-0.394	0.040		0.359	0.052	0.022	0.189	0.007	0.202
Non-HDL-C	-0.362	-0.080	-0.297	-0.138	-0.148	0.583	0.553	0.359	0.829	-0.535	0.914	0.359		-0.367	-0.218	0.203	-0.050	0.113
BoP(%)	0.145	0.193	-0.069	-0.036	-0.077	-0.102	-0.158	0.016	-0.271	0.140	-0.387	0.052	-0.367		-0.056	-0.168	-0.174	-0.085
PD 4-5 mm	-0.014	-0.216	-0.043	0.255	-0.127	-0.200	-0.141	0.035	-0.164	0.162	-0.193	0.022	-0.218	-0.056		-0.041	0.526	0.457
PD ≥6 mm	-0.247	-0.139	0.350	0.392	-0.056	0.028	0.056	0.184	0.147	-0.217	0.196	0.189	0.203	-0.168	-0.041		0.147	0.324
CAL 3-4 mm	0.198	-0.013	-0.129	0.171	0.067	0.020	0.084	0.044	-0.040	0.004	-0.041	0.007	-0.050	-0.174	0.526	0.147		0.550
CAL≥ 5mm	0.129	0.100	-0.152	0.228	-0.256	0.173	0.176	0.254	0.044	-0.157	0.099	0.202	0.113	-0.085	0.457	0.324	0.550	

SOD1= Superoxide dismutase 1; *IRS2*= Insulin Receptor Substrate 2; HbA1c= Glycated hemoglobin; HOMA= Homeostasis model assessment; BMI= Body mass index; VLDL= Very low-density lipoprotein; BoP= Bleeding on probing; PD= Probing depth; CAL= Clinical attachment level. Values above light gray meant p values. The green colors meant that there was a positive significant correlation, and the red color meant negative significant correlation. Upper diagonal= p values. Lower diagonal values= p= Spearman's correlation coefficients.
